# Entangled time in flocking: Multi-time-scale interaction reveals emergence of inherent noise

**DOI:** 10.1371/journal.pone.0195988

**Published:** 2018-04-24

**Authors:** Takayuki Niizato, Hisashi Murakami

**Affiliations:** 1 Tsukuba University, Faculty of Engineering, Information and Systems, Tsukuba, Ibaraki, Japan; 2 University of Tokyo, Research Center for Advanced Science and Technology, Megro, Tokyo, Japan; Abdus Salam Centro internazionale di fisica teorica, ITALY

## Abstract

Collective behaviors that seem highly ordered and result in collective alignment, such as schooling by fish and flocking by birds, arise from seamless shuffling (such as super-diffusion) and bustling inside groups (such as Lévy walks). However, such noisy behavior inside groups appears to preclude the collective behavior: intuitively, we expect that noisy behavior would lead to the group being destabilized and broken into small sub groups, and high alignment seems to preclude shuffling of neighbors. Although statistical modeling approaches with extrinsic noise, such as the maximum entropy approach, have provided some reasonable descriptions, they ignore the cognitive perspective of the individuals. In this paper, we try to explain how the group tendency, that is, high alignment, and highly noisy individual behavior can coexist in a single framework. The key aspect of our approach is multi-time-scale interaction emerging from the existence of an interaction radius that reflects short-term and long-term predictions. This multi-time-scale interaction is a natural extension of the attraction and alignment concept in many flocking models. When we apply this method in a two-dimensional model, various flocking behaviors, such as swarming, milling, and schooling, emerge. The approach also explains the appearance of super-diffusion, the Lévy walk in groups, and local equilibria. At the end of this paper, we discuss future developments, including extending our model to three dimensions.

## Introduction

Collective behavior, such as swarming, schooling and flocking, is widely observed in nature [1–3]. These highly ordered behaviors of a group have been compared to a single body with one mind [4]. The emergence of these behaviors has been investigated by computer simulations, such as the Boids model and the self-propelled particle model [5–9]. In these models, each agent in has neighbors within a certain radius or ranking distance (topological distance) [10–13], and agents coordinate their behavior with these neighbors. This mutual coordination with some extra interactions produces various flocking formations.

However, experimental studies show that within the coherent collective behavior, the behavior of individuals seems disordered [14–18]. In starling flocks, the neighbors of each bird shuffle and are not fixed [17]. Super-diffusion in collective behavior supports this observation. The diffusion of birds in flocks is faster than Brownian motion, and this super-diffusion is also observed in fish schools (e.g., *Plecoglossus altivelis*) [19]. Murakami et al. also found that, when described in the center of a mass reference frame, individuals’ trajectories in fish schools show Lévy walks, which are an optimal strategy for balancing exploration and exploitation, [20, 21]. Furthermore, their results suggest the trajectory of each fish is not constrained to a local region of the group but covers the whole group.

It has been pointed out that these noisy behaviors facilitate the collective behavior [22, 23]. For instance, locusts in a bounded condition adjust their noisy behavior according to environment [14] and large swarms of midges use their correlation (the degree of how much and how far the individual’s behavior affects the others it is not directly interacting with) to achieve collective behavior despite the lack of collective order [22, 23]. The correlation in the group enlarges each individual’s effective perception range so that it is larger than its interaction range. This enlarged perception range increases the group’s susceptibility to external perturbation [24, 25]. Recently, Mateo et al. [26] analyzed the correlation effect in terms of network structures in flock simulations.

However, the correlation itself does not explain how individuals in flocks generate their noise-like behavior, because the correlation in the flock only tells us how the information (individual’s perturbation) is spread and shared with other members, and never tells us how an individual’s decision in the group will be made in a certain environment. For instance, the study of locust swarms suggests that each locust tunes its noisy behavior by itself according to its local polarity [14]. Self-tuning noisy behavior is also observed in *Plecoglossus altivelis* schools more directly, since the trajectory of the individual fish in the center of the mass reference shows motion that is sometimes ballistic and at other times entangled [19]. All these studies suggest that these self-regulated noisy movements inside the group need to be explained independently with correlational properties because the statistical analysis lacks individual’s perspective. Until now, there have been few studies that have tried to explain these behaviors [27].

Before we describe our flocking model, we begin by discussing the origin of alignment and attraction over multiple time scales. The attraction and alignment forces used in most models result from the positions and directions of an agent’s neighbors. The balance between these two forces is important for producing various flocking formations, such as swarming, milling, and schooling [5, 28–41].

Each agent in a flock is attracted to its neighbor’s current position and aligns its direction of motion to that of its neighbor. From a conventional viewpoint, attraction depends on the neighbors’ positions, whereas alignment depends on the neighbors’ directions of motion [2]. However, alignment also means that each agent is attracted to the infinite-future position of its neighbor along the neighbor’s current direction of motion. Thus the difference between alignment and attraction can be viewed as lying not in their properties (direction vs. position) but in the time scale, that is, attraction to the current position (*t* = 0) vs. attraction to the infinite-future position (*t* = ∞) of a neighbor. Thus, attraction and alignment are interactions on two extreme time scales. If agents have various time scales between these two extremes, we should expect that various flocking behaviors to emerge.

By considering these multi-time-scale interactions, we constructed a new flocking model that shows various flocking behaviors, such as swarming, milling, and schooling, which can coexist. The parameters of our model do not need to be tuned, unlike previous models. Furthermore, if we modify some interactions in our model, we can also obtain self-propelled particle-like behavior. This proves that our model is a natural extension of previous models.

The coexistence of various flocking formations means that flocking behaviors in our model are built on the noisy movement of individuals, which has statistical regularities, such as super-diffusion and Lévy walks in groups. In addition, we also demonstrate that our flocking model is at local equilibrium; thus, the local alignment speeds are much faster than the network arrangement speeds [42]. Our model reproduces all these empirical results.

Finally, we discuss future developments for our model, including extending it to three dimensions and using other interaction formats.

## Material and method

### Origin of alignment and attraction

First, we provide a brief overview of alignment and attraction in flocking models. Although alignment and attraction have been widely used in many flocking models, there have been few discussions of what alignment and attraction mean. Alignment is the coordinating of the direction of motion with an agent’s neighbors and attraction is the tendency of the agent to move toward a neighbor’s current position. Some studies suggest that alignment has a weak effect in fish schools [31–33]. This may be because, in the captive environments in which fish schools are usually observed, the concept of infinite-future positions may not be appropriate. However, for starling flocks, alignment fits well with the empirical results because birds have effectively infinite space in the sky [15–17].

**Fig 1:**
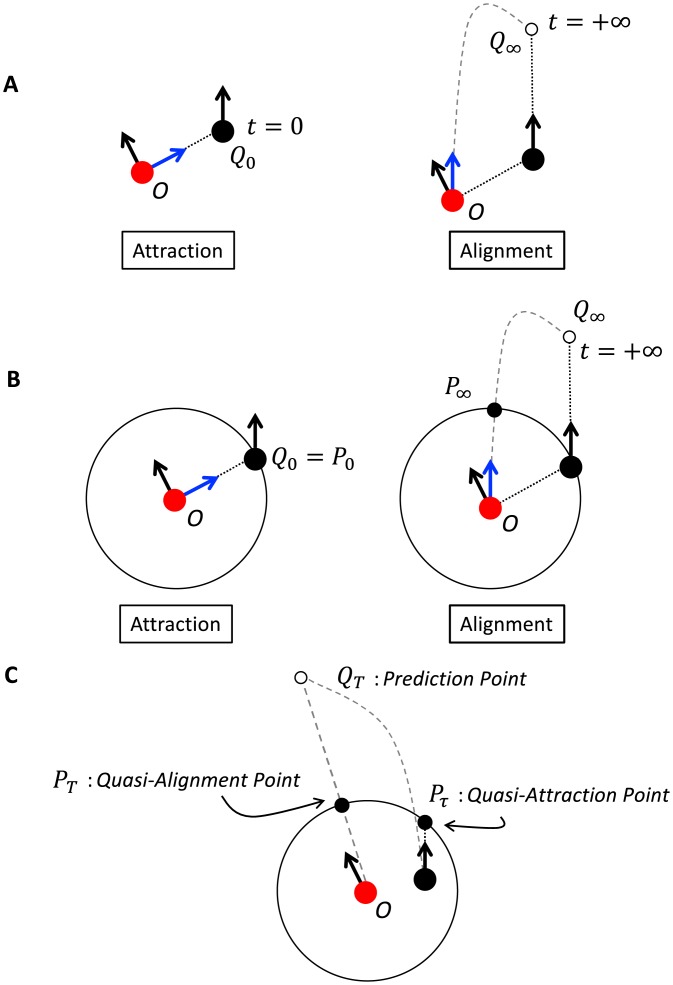
Schematics of attraction, alignment, and their generalization. (A) Schematic of attraction (left) and alignment (right). The agent (red dot) is attracted toward its neighbor’s (black dot) current position (*Q*_0_: left) and infinite future position (*Q*_∞_: right) (B) Schematic of attraction and alignment with a neighborhood. The circle indicates the boundary of the agent’s neighborhood. In the attraction case (left), the point *P*_0_ is precisely the same as *Q*_0_. In the alignment case (right), the point *P*_∞_ reflects the information of *Q*_∞_ as the point on the boundary the agent would reach if it moved in a direction parallel with its neighbor’s motion. (C) Schematic of the generalization from *P*_0_ and *P*_∞_ to *P*_*τ*_ and *P*_*T*_. *P*_*τ*_ is the crossing point of the agent’s extended direction vector at the boundary of the neighborhood. *P*_*T*_ is the point where the vector from the agent to a certain prediction point *Q*_*T*_ crosses the boundary of the neighborhood.

Although attraction and alignment seem quite different because of the difference between the properties on which they act, we can describe alignment in a way that is similar to the description of attraction. If an agent adjusts its direction of motion to that of its neighbor, the agent points toward the infinite-future position of its neighbor (see Fig 1A; note that the dashed line connecting the agent of interest at *O* to the infinite-future position of its neighbor is only curved because of the constraint of representing a point at infinity on a finite page). Alignment is, therefore, the result of a kind of attraction: “attraction” is an attraction to the neighbor’s current position, *Q*_0_, while “alignment” is an attraction to the infinite-future position of the neighbor, *Q*_∞_. Thus, the difference between alignment and attraction is not in the interaction properties but in the time scales.

### Short-term and long-term prediction on the edge of agent’s neighborhood

These two time scales hint at other possible flocking models. Taking this view of attraction, allows us to consider attraction at arbitrary time scales *t* between the two extremes *Q*_0_ and *Q*_∞_, namely, attraction to *Q*_*t*_. To implement these intermediate points of attraction in our model, we convert each point *Q*_*t*_ to a point *P*_*t*_ on the edge of an agent’s neighborhood. In Fig 1B a circular neighborhood is superimposed upon the diagrams in Fig 1A. Attraction point *P*_0_ is the position of a neighbor on the edge of an agent’s neighborhood, and *P*_∞_ is the point that the agent’s neighborhood intersects with the dashed line from *O* to *Q*_∞_ (*O* is the position of the agent of interest). The two extremes *Q*_0_ and *Q*_∞_ are thus converted to points on the edge of the neighborhood as *P*_0_ and *P*_∞_, respectively. Note that attraction point *P*_0_ is derived from information *Q*_0_ from inside the neighborhood and alignment point *P*_∞_ is derived from information *Q*_∞_ from outside the neighborhood.

Fig 1C shows a generalization of our discussion. There is one neighbor within the agent’s neighborhood. We define the quasi-attraction point *P*_*τ*_ as the point where the extended neighbor’s direction vector crosses the agent’s neighborhood. When the neighbor is near the edge of the neighborhood, the quasi-attraction point is almost the same as the attraction point. Next, the quasi-alignment point *P*_*Τ*_ is defined as the intersection point where the vector to a prediction point (white circle in Fig 1C) from the center crosses the boundary of the agent’s neighborhood. The type of prediction determines the flocking behavior. In this paper, we use the alignment prediction and anticipation methods. Note that *τ* and *T* represent the two most extreme prediction times. *T* is always larger than *τ* because *τ* is related to a short-term prediction while *T* is related to a long-term prediction: a neighbor would only take a short time (minimum time of zero) to reach the edge of the agent’s neighborhood from the inside while it would take longer to reach a point outside of the neighborhood (e.g., the white circle in Fig 1C). Not also that, since *Q*_*T*_ and *P*_*τ*_ are only points on a neighbor’s predicted trajectory, the neighbor need not actually travel to either of these points. However, one implication of these predictions should be noted. Through the generalization process shown in Fig 1C, the relation of the two extremes *P*_0_ and *P*_∞_, which we have observed, is weakened while preserving its original properties.

### Prediction methods

The time scales *τ* and *Τ* can take many values according to the neighbor’s position relative to the boundary of the agent’s neighborhood and the prediction method. In this section, we introduce the alignment prediction and anticipation methods. There may be other prediction methods; however, these are the only examples that we have found. In this paper, we only use the anticipation method for our analysis. The alignment method is only used to show that this method can create self-propelled particle-like behavior.

**Fig 2:**
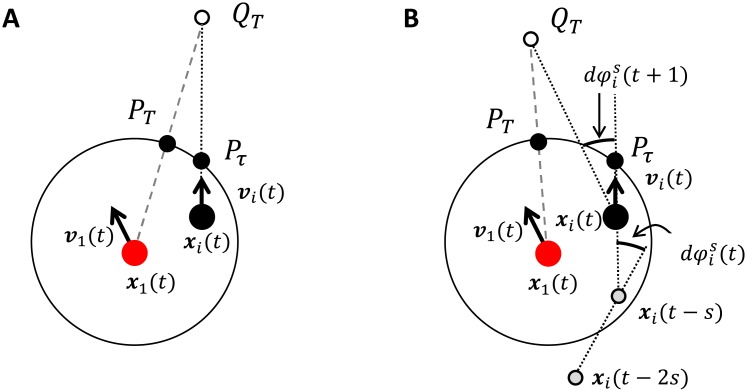
Schematics of the two prediction methods. (A) Alignment prediction: the prediction point *Q*_*T*_ is the end point of the elongated neighbor’s direction vector ***v***_*i*_(*t*). (B) Anticipation: the prediction point *Q*_*T*_ is the predicted landing point if the turning rate of the agent’s neighbor is the same as its previous rate, that is, *dφ*_*i*_^*s*^(*t*) = *dφ*_*i*_^*s*^(*t +* 1). The value of *s* indicates how long the agent refers to its neighbor’s past movement in terms of a number of time steps. The quasi-alignment point *P*_*T*_ is the point where the segment from the agent to the prediction point *Q*_*T*_ crosses the boundary of the neighborhood.

Alignment prediction: This prediction method is similar to the alignment interaction that is widely used. This method produces group behaviors like the self-propelled particle model (S1 Movie). Fig 2A shows a schematic of the alignment prediction method. The end point of an extremely elongated neighbor’s direction vector ***v***_*i*_ is used as the prediction point *Q*_*T*_ (white circle in Fig 2A). Thus, the prediction point is *Q*_*T*_ = ***x***_*i*_(*t*) + *T****v***_*i*_(*t*), where ***x***_*i*_(*t*) is neighbor *i*’s position and ***v***_*i*_(*t*) is its velocity vector at time *t*. In this paper, we fixed *T* = 300 because this method is used only to provide an example of the generation of self-propelled-particle-model-like behavior.
Anticipation: This prediction method is similar to that of Morin et al. [35). Past direction turning rate *dφ*_*i*_(*t*) affects the future direction turning rate *dφ*_*i*_(*t +* 1), assuming *dφ*_*i*_(*t+*1) = *dφ*_*i*_ (*t*) for the prediction (Fig 2B). For precision we write *dφ*_*i*_(*t*) as *dφ*_*i*_^*s*^(*t*) because the degree of *dφ*_*i*_ depends on how long the agent of interest refers to its neighbor’s past movements (*s* steps), that is, *dφ*_*i*_^*s*^(*t*) = arg(***v***_*i*_^*s*^(*t*)) − arg(***v***_*i*_^*s*-1^(*t*)) where ***v***_*i*_^*s*^(*t*) = ***x***_*i*_(*t*) − ***x***_*i*_(*t*−*s*) and ***v***_*i*_^*s*-1^(*t*) = ***x***_*i*_(*t*−*s*) − ***x***_*i*_(*t*−2*s*). The prediction point for anticipation is *Q*_*T*_ *=* ***x***_*i*_(*t*) + *T* ***|| v***_*i*_(*t*) ***|| u***_*i*_(*t*), where ***u***_*i*_(*t*) is a unit vector with argument arg(***v***_*i*_^1^(*t*)) + *dφ*_*i*_(*t*). The symbol ||-|| indicates the vector norm. The value of *T* is given by s*r_1_(t)/ ***|| v***_*i*_(*t*) ***||*** where *r*_1_(*t*) is the radius of the neighborhood at time *t*. This definition means that enlarging *s*, that is how long the agent refers to its neighbor’s past, enlarges *T*. The value of *s* is uniquely determined as the minimum value such that all neighbors’ *Q*_*T*_*’*s lie outside the neighborhood. In S1 Fig, we give the graph of the distribution of *s*, which shows the frequency of *s* values for our simulations. We use this method for all the results (S2 Movie).

### Method descriptions

In this section, we describe our flocking model algorithm. The parameter settings and other more complicated mathematical expressions are provided in the Method section and S1 Text.

First, we define the functions and symbols that we use in our model. We define four functions: (i) The distance function *d*(***x***, ***y***); its output is the Euclidean distance between position vectors ***x*** and ***y*** in ***R***^*n*^ (in this paper, *n* = 2). (ii) The mean angle function mean(*X*); its output is the mean angular direction in set *X*. (iii) The covering function Cov(*X*); its output is the set {*θ*| min({*θ*_*i*_ − mean(*X*)| *θ*_*i*_ ∈ *X* }) ≤ *θ* − mean(*X*) ≤ max({*θ*_*i*_ − mean(*X*)| *θ*_*i*_ ∈ *X* })}. The relation *X* ⊆ Cov(*X*) always holds. (vi) The random function *Ran*(*S*): its output is an element *x* randomly selected from set *S*.

Next, we define four sets for our model. ***N*** = {1, 2, …, *N*} are the tags for the agents and *N* = |***N***|, where |-| indicates the cardinality of a set. ***E*** = {(*i*, *j*)| *i*, *j* ∈ ***N***, *i* and *j* are Voronoi neighbors} is a network constructed from all directly connected Delaunay triangles. The set ***n***_*i*_ = {*j*| *j* ∈ ***N***, (*i*, *j*) ∈ ***E*** or *i* = *j*} represents all of agent *i*’s neighbors, including itself. The set ***o***_*i*_ = {*j*| *j* ∈ ***N***, *d*(***x***_*i*_, ***x***_*j*_) < *R* or *i* = *j*} represents all of agent *i*’s neighbors inside its repulsion zone, including itself. *R* is a parameter giving the size of the repulsion zone, which is fixed in our simulations. Our model uses the Voronoi neighborhood to determine neighbors because recent studies, especially in fish schools, suggest that the Voronoi neighborhood provides a more valid description of collective behavior than other neighborhoods [36, 37].

We need to define a circular neighborhood including all Voronoi-connected-neighbors ***n***_*i*_(*t*) for agent *i* at time *t* to obtain quasi-attraction points (i.e., *P*_*τ*_*’*s) and quasi-alignment points (i.e., *P*_*T*_*’*s) on the boundary of this neighborhood. This circle is *C*_*i*_(*t*) = ***x***_*i*_(*t*) + {(*r*_*i*_(*t*)cos(*θ*), *r*_*i*_(*t*) sin(*θ*))| 0 ≦ *θ*<2π} where *r*_*i*_(*t*) = max({*d*(***x***_*i*_(*t*), ***x***_*j*_(*t*)) | *j* ∈ ***n***_*i*_(*t*)}) + *c* (*c* is a constant parameter). All short-term (i.e., *P*_*τ*_) and long-term (i.e., *P*_*T*_) predictions for an agent *i* lie on this circle *C*_*i*_(*t*).

**Fig 3:**
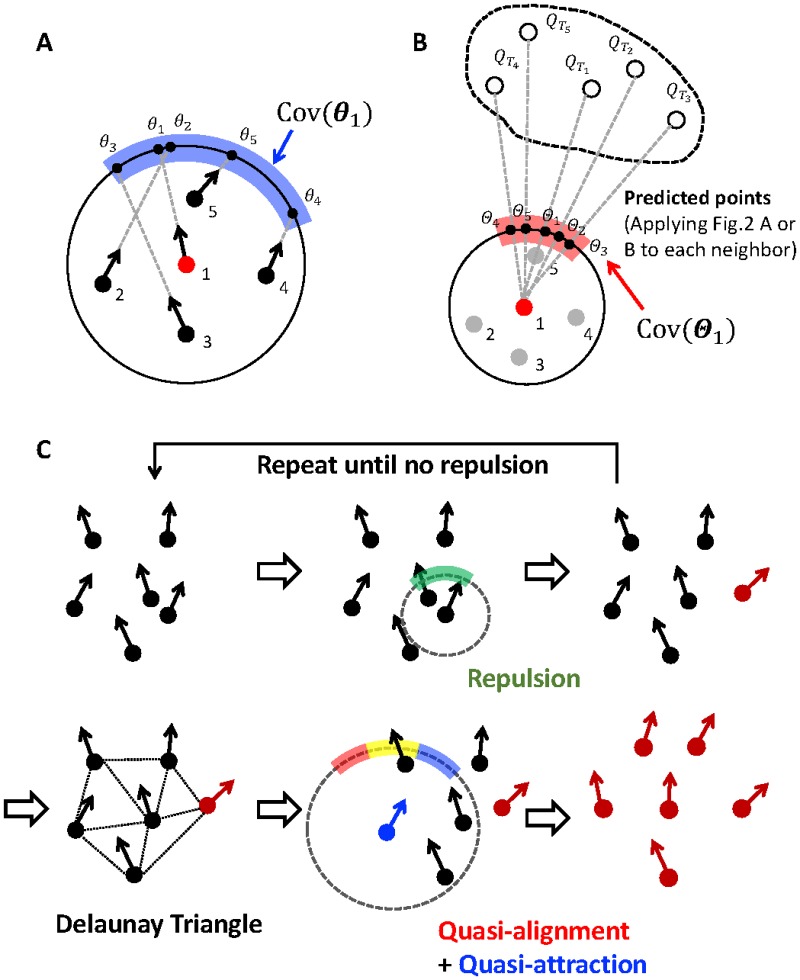
Schematics of the showing short-term and long-term prediction on the circle. (A) The circle shows quasi-attractions as internal information for agent 1. The numbers under the dots are the tags for the agents. The bold blue arc is a covering set for ***θ***_1_. (B) The circle shows quasi-alignments as external information for agent 1. Each prediction point $Q_{T_{i}}$ determines *Θ*_i_. The bold red arc is a covering set for ***Θ***_1_. (C) The complete chart of our algorithm. In the first step, find an agent which has its neighbors within its repulsion range ($\text{Cov(}{\tilde{\theta }}_{i}\left(t\right)$) colored green) and apply the repulsion algorithm (the updated agent is shown in red). Repeat this until there is no agent which has its neighbors within its repulsion range. Then, construct a Delaunay triangulation from all agents’ positional information. Apply quasi-attraction (Cov(***θ***_*i*_(*t*)) colored blue) and quasi-alignment (Cov(***Θ***_*i*_(*t*)) colored red) and take their intersection (*I*_*i*_(*t*) colored yellow). The agent of interest (colored blue) selects its direction from this yellow interval.

Here, we describe the two interaction methods (Fig 3A and 3B).

1. *Direction through common information*: A quasi-attraction point collection of *P*_*τ*_*’*s for *i* is a collection of directions, ***θ***_*i*_(*t*) = {*θ*_1_(*t*), *θ*_2_(*t*), …, *θ*_***|n****i*(*t*)*|*_ (*t*)}, because only directional information matters for *C*_*i*_(*t*) in our model (Fig 3A). Similarly, a quasi-alignment point collection of *P*_*T*_s for *i* is also a collection of directions, ***Θ***_*i*_(*t*) = {*Θ*_1_(*t*), *Θ*_2_(*t*), …, *Θ*_***|n****i*(*t*)*|*_ (*t*)} (Fig 3B). Each *P*_*T*_ is given from *Q*_*T*_ using the prediction method, that is, alignment or anticipation (see the section *Prediction Methods*). We take the intersection between Cov(***θ***_*i*_(*t*)) and Cov(***Θ***_*i*_(*t*)), *I*_*i*_(*t*) = Cov(***θ***_*i*_(*t*)) ∩ Cov(***Θ***_*i*_(*t*)). These common short-term (i.e., *P*_*τ*_) and long-term (i.e., *P*_*T*_) predictions determine agent *i*’s next direction of motion, *φ*_*i*_(*t +* 1) = mean(*φ*_*i*_(*t*), *Ran*(*I*_*i*_(*t*))). In particular, in the case *I*_*i*_(*t*) = ∅, *φ*_*i*_(*t +* 1) = mean(*φ*_*i*_(*t*), *Ran*(*C*_**u**_—*J*_*i*_(*t*))), where *J*_*i*_ (*t*) = Cov(***θ***_*i*_(*t*)) ∪ Cov(***Θ***_*i*_(*t*)) and *C*_**u**_ is a unit circle.
2. *Repulsion*: Repulsion in our model is a deflection of an agent’s movement direction from the interval. Because the neighbors in its repulsion zone are ***o***_*i*_, a quasi-attraction point collection of *P*_*τ*_ on ${\tilde{C}}_{i}\left(t\right)=x_{i}\left(t\right)+\{(R\;\text{cos}(\theta ),R\;\text{sin}(\theta ))|0≦\theta <2\pi \}$ gives ${\tilde{\theta }}_{i}\left(t\right)=\left\{\theta_{1}\left(t\right),\theta_{2}\left(t\right),\;\ldots ,\theta_{|o}{{}_{i}}_{\left(t)\right|}\left(t\right)\right\}$. Then, agent *i*’s next direction is $\varphi_{i}\left(t\;+1\right)=\text{mean}(\varphi_{i}\left(t\right),Ran(C_{u}–\text{Cov(}{\tilde{\theta }}_{i}\left(t\right)\text{)))}$, where *C*_**u**_ is a unit circle.

The intersection *I*_*i*_(*t*) in (I) is constructed from both short-term predictions (i.e., quasi-attractions) and long-term predictions (i.e., quasi-alignments) on the circle *C*_*i*_. Each agent can choose any direction from the interval *I*_*i*_(*t*). The interaction in our model only determines a bundle of directions in an obtained interval, but not a certain direction. This fact suggests that the direction that the agent chooses always has some uncertainty, even if it can precisely compute and predict all its neighbors’ behaviors, because the agent can only obtain interval information *I*_*i*_(*t*) and select one element from this interval.

#### Noise definition

In this paper, we regard noise as arising from an agent’s ability to resolve space divisions (i.e., a cognitive resolution of space). The bundle of directions in interval *I* gives the next direction of motion for an agent. Treating noise as cognitive resolution blurs this interval (see S2 Fig). This assumption comes from the finite nature of the accuracy of animal cognitive systems (e.g., animal visual perception) [38–41].

Consider the *p*-division of unit circle *C*_**u**_ for agent *i*, where *p* is a natural number. The *p*-division acts on circle *C*_**u**_ centered on the agent’s position at time *t*. Each fragment of the circle is ${\tilde{c}}_{a}\;=\;\{\theta |\varphi_{i}(t)+\frac{2\pi }{p}a\leq \theta <\varphi_{i}(t)+\frac{2\pi }{p}(a+1)\}$, where the agent direction of motion is *φ*_*i*_(*t*) and 0 ≦ *a*<*p*. Note that ${\tilde{c}}_{a}\cap {\tilde{c}}_{b}\;=\;\emptyset$ if *a*≠*b* and $C_{u}\;=\;{⋃}_{a\;=\;0}^{p-1}{\tilde{c}}_{a}$. The size of each fragment, ${\tilde{c}}_{a}$, indicates the ability of the agent to divide space (i.e., the degree of directional resolution). Thus, intervals *I* can be partitioned into ${⋃}_{a\in [I]}{\tilde{c}}_{a}$, where $\left[I\right]=\{a|0≦a<p,\;{\tilde{c}}_{a}\cap I\neq \emptyset \}$. Applying this method, we can rewrite Eqs. (I) and (II) as follows.

$$\begin{array}{ll} \left(\text{I}\prime \right):\;\;\varphi_{i}\left(t+1\right) & =\text{mean}\left(\varphi_{i}\left(t\right),\;Ran\left({\tilde{c}}_{Ran(I_{i}\left(t\right)\})}\right)\right)\text{or}\;\text{mean}\left(\varphi_{i}\left(t\right),\;Ran\left({\tilde{c}}_{Ran\left(\left[C_{u}-J_{i}\left(t\right)\right]\right)}\right)\right)\\ \left(\text{II}\prime \prime \right):\;\varphi_{i}\left(t+1\right) & =\text{mean}\left(\varphi_{i}\left(t\right),Ran\left({\tilde{c}}_{Ran\left(\left[C_{u}-\text{Cov}\left({\tilde{\theta }}_{i}\left(t\right)\right)\right]\right)}\right)\right) \end{array}$$

Large *p* preserves the original structure of *I*. Interval *I* and partitioned interval $\text{Cov}\left(I\right)=\cup_{a\in \left[I\right]}{\tilde{c}}_{a}$ are generally equal. However, when *p* is small, the partitioned interval $\cup_{a\in \left[I\right]}{\tilde{c}}_{a}$ always includes the original interval *I*. This mismatch generates more choices from Cov(*I*) than from the original interval *I* because the selection area from which each agent chooses a direction expands (S2 Fig). In this paper, a noise parameterζdetermines the value of $p\;=\;[\frac{2\pi }{\zeta }]$, where [–] is the floor function. Note that noise strength is not the same as used in traditional models. Noise in traditional models acts on an agent’s pre-determined direction of motion. On the other hand, the role of noise in our model is only to expand the interval Cov(*I*) and increase the set of directions which the agent can take.

#### Speed definition

We consider that turning rate curbs the agent’s speed (for instance, in [19] *Plecoglossus altivelis*’s speed is small when the turning rate is large). So, we define agent *i*’s speed at time *t* as ***v***_*i*_(*t* + 1) = *V*cos(*φ*_*i*_(*t +* 1)–*φ*_*i*_(*t*)) where *V* is a maximum velocity.

### Algorithm

1. Distribute all agents randomly in a two-dimensional space, which is 100(*m*)×100(*m*) in our model. Each agent also has a random velocity.
2. Each agent checks whether it has its neighbors in its repulsion zone ${\tilde{C}}_{i}(t)\neq \emptyset$ or not. If it does, go to 2.1. Otherwise, go to 3.
  2.1. Compute the agent’s quasi-attraction points on ${\tilde{C}}_{i}\left(t\right)=x_{i}\left(t\right)+\{R\text{cos}(\theta ),R\text{sin}(\theta ))|0≦\theta <2\pi \}$ for each agent in ***o***_*i*_(*t*) = {*j*|*d*(***x***_*i*_(*t*), ***x***_*j*_(*t*)) < *R*} to obtain ${\tilde{\theta }}_{i}\left(t\right)=\left\{\theta_{1}\left(t\right),\theta_{2}\left(t\right),\ldots ,\theta_{|o}{{}_{i}}_{\left(t)\right|}\left(t\right)\right\}$. Make a covering set, $\text{Cov}({\tilde{\theta }}_{i}\left(t\right))$, and determine the agent’s next direction, $\varphi_{i}\left(t\;+1\right)=\text{mean}(\varphi_{i}\left(t\right),Ran({\tilde{c}}_{Ran\left(\left[C_{u}-\text{Cov}\left({\tilde{\theta }}_{i}\left(t\right)\right)\right]\right)}))$.
  2.2. Determine the agent’s velocity: ***v***_*i*_(*t* + 1) = *V*cos(*φ*_*i*_(*t +* 1)–*φ*_*i*_(*t*)) where *V* is a maximum velocity.
  2.3. Update the agent’s position: ***x***_*i*_(*t* + 1) = ***x***_*i*_(*t*) + ***v***_*i*_(*t* + 1).
3. Draw Delaunay triangles (i.e., find the Voronoi neighbors) ***E***(*t*) = {(*i*, *j*)| *i*, *j* ∈ ***N***, *i* and *j* are Voronoi neighbors} from agent’s distribution to find its directly connected neighbors, ***n***_*i*_(*t*) = {*j*| *j* ∈ ***N***, (*i*, *j*) ∈ ***E***(*t*) or *i* = *j*}. Obtain the edge of neighborhood *C*_*i*_(*t*) = ***x***_*i*_(*t*) + {(*r*_*i*_(*t*)cos(*θ*), *r*_*i*_(*t*) sin(*θ*))| 0 ≦ *θ*<2π} where *r*_*i*_(*t*) = max({*d*(***x***_*i*_(*t*), ***x***_*j*_(*t*)) | *j* ∈ ***n***_*i*_(*t*)}) + *c* (*c* is a constant parameter). Compute the agent’s quasi-attraction and quasi-alignment points for ***n***_*i*_(*t*) and get ***θ***_*i*_(*t*) and ***Θ***_*i*_(*t*) by using the method (I). Make two covering sets (Cov(***θ***_*i*_(*t*)) and Cov(***Θ***_*i*_(*t*))) and take the intersection, *I*_*i*_(*t*) = Cov(***θ***_*i*_(*t*)) ∩ Cov(***Θ***_*i*_(*t*)).
  3.1. If *I*_*i*_(*t*) ≠ ∅, the agent’s next direction is $\varphi_{i}\left(t\;+1\right)=\text{mean}(\varphi_{i}\left(t\right),Ran({\tilde{c}}_{Ran\left(\left[I_{i}\left(t\right)\right]\right)}))$.
  3.2. If *I*_*i*_(*t*) = ∅, the agent’s next direction is $\varphi_{i}\left(t\;+1\right)=\text{mean}(\varphi_{i}\left(t\right),Ran({\tilde{c}}_{Ran\left(\left[C_{u}-J_{i}\left(t\right)\right]\right)}))$
4. Update the rest of the agent states ***v***_*i*_(*t* + 1) = *V*cos(*φ*_*i*_(*t +* 1)–*φ*_*i*_(*t*)) and ***x***_*i*_(*t* + 1) = ***x***_*i*_(*t*) + ***v***_*i*_(*t* + 1) synchronously. Then update *t* → *t* + 1 and return to 2.

In Fig 3C, we describe the chart of our algorithm. The parameters are listed in the Method section. This model has no boundary conditions. Almost all agents are simultaneously updated by using the three steps; however, some agents apply step 2 before proceeding. This procedure is introduced to avoid the situation of agents’ positions coinciding. Thus, our model uses a semi-synchronous update rule.

## Results

### Schooling, milling, and swarming

First, we examine the effects of noise as cognitive resolution (the ability to divide space; in this case, circle division) and the maximum velocity *V* on the collective behavior of our model. To summarize the agents’ behavior, we use two measures, the degree of polarity *O*_*P*_ and torus *O*_*T*_, which have been widely used for collective behavior. High polarity *O*_*P*_ means that the groups are in high alignment (ordered state) and high *O*_*T*_ means that groups are in a milling state, which is sometimes observed in fish schools. In contrast, low *O*_*P*_ and *O*_*T*_ values indicate swarming (i.e., the flock is in a completely disordered state). Note that 0 ≦ *O*_*P*_, *O*_*T*_ ≦1. *O*_*P*_ and *O*_*T*_ are expressed mathematically as

$$O_{P}\left(t\right)=\frac{1}{N}\left|\sum_{i=1}^{N}u_{i}\left(t\right)\right|,$$ (1)

$$O_{T}\left(t\right)=\frac{1}{N}\left|\sum_{i=1}^{N}u_{i}\left(t\right)\times q_{i}\left(t\right)\right|,$$ (2)

where *N* is the number of agents (|***N***| = *N*), ***u***_*i*_ is the unit velocity vector of agent *i*, and ***q***_*i*_ is a unit vector pointing from the group’s center of mass toward agent *i*. Most flocking models have suggested that these qualitatively different behaviors (schooling, milling, and swarming) are parameter-dependent, which means a certain set of parameters corresponds to each group’s behavior. The parametric region for producing multiple states at the same time is a restricted one [37].

**Fig 4:**
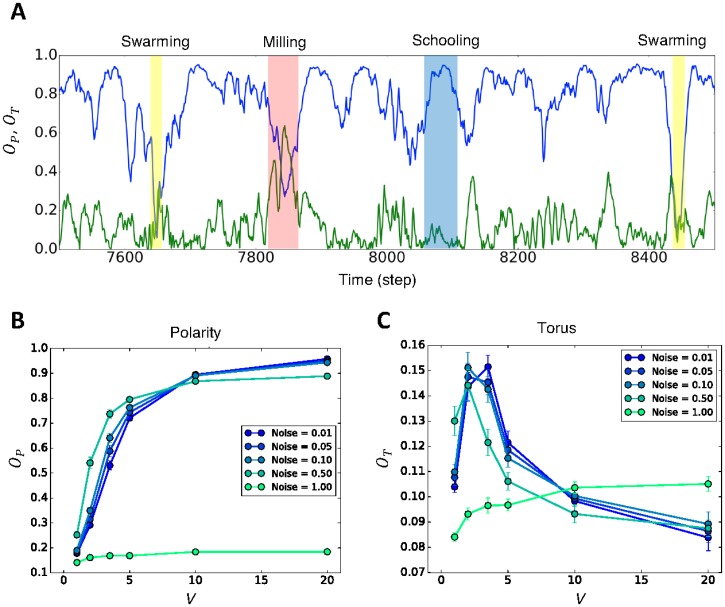
Averaged polarity *O*_*P*_ and torus *O*_*T*_ values and their time series. (A) Time series of *O*_*P*_ and *O*_*T*_ values (*N* = 100). (B) Polarity for the conditions *V* = 1.0, 2.0, 3.5, 5.0, 10.0, and 20.0 with respect to noise of 0.01, 0.05, 0.10, 0.50, and 1.00. *V* is the maximum velocity that each agent can have. The graph shows low polarity in the low-velocity regions because of repulsion effects as we discussed in the main text. (C) Torus structure for the conditions *V* = 1.0, 2.0, 3.5, 5.0, 10.0, and 20.0 with respect to noise of 0.01, 0.05, 0.10, 0.50, and 1.00 (628, 124, 62, 12, and 6 divisions of the unit circle). The 17,000 steps were run 30 times for all cases.

However, Fig 4A suggests that agents in our model (*N* = 100) can switch between schooling (high alignment), milling, and swarming behaviors. Agents can have a high *O*_*P*_, a high *O*_*T*_, or a low *O*_*P*_ and *O*_*T*_ (S2 Movie). Almost all parameter settings (velocity and noise intensity) have the same tendency in terms of the coexistence of three different behaviors (Fig 4B and 4C). Even if the agents are in a highly ordered state on average (*O*_*p*_>0.9), their minimum *O*_*P*_ is lower than 0.3 in most cases (S3 Fig). This fact suggests that high alignment flocks are not always stable. Although milling states are observed more often in low-velocity regions (S3 Fig), high *O*_*T*_ values above 0.5 remain in both the high-velocity and noise regions. To achieve various collective behaviors in flock models, many researchers have suggested that the balance between attraction and alignment is crucial. In contrast, the agents in our model can tune the balance between attraction and alignment as the time scales (*τ* and *T*) by themselves, as discussed in the previous section.

Noise as cognitive resolution works differently compared with past flocking models. Usually, increasing the noise intensity results in disordered groups. Although this trend is also observed for high noise intensity (light green in Fig 4B), it is not always the case. In low-velocity regions, noise increases the *O*_*P*_ on average.

In the low-velocity region, the agents collide with each other because agents tend to stick together. Collisions happen less frequently in the high-velocity case. It is true that each agent tends to move closer in this model, but high velocity decreases the possibility of becoming trapped in another agent’s repulsion region because high-velocity agents pass through other agents’ repulsion areas (in our simulation, the average collision rate in a group of 100 agents per step is 4.54, 1.49 and 0.77 for *V*/*R* = 4/4, 7/4 and 10/4, respectively). These collisions destabilize the flock alignment.

The effect of this misalignment by collision would be greater for relatively low noise intensity than for relatively high noise intensity. Indeed, low noise agents show high alignment, but the unexpected large direction changes (repulsion in this case) are easily spread all over the flock because of the high space division ability. This perturbation spreading tendency prevents low noise agents from creating high alignment in the low-velocity region.

### Diffusion in flocks

Recent findings about real flocks and schools have shown that individual behavior inside the group is disordered rather than highly ordered. Seamless shuffling with neighbors and bustling movements have been little reported in flocking models [11]. In particular, the maximum entropy approach is known to be able to describe these collective properties well, but it does not provide individual perspectives to describe what is going on inside these collective behaviors. Murakami’s recent finding on the Lévy walks inside fish schools shows the statistical regularity in these movements [19]. Exploring and exploiting the behavior of each single fish in the school suggest individual intelligent behavior within the group. It is difficult to understand their finding without considering each individual’s decision under imperfect and fragmented information.

**Fig 5:**
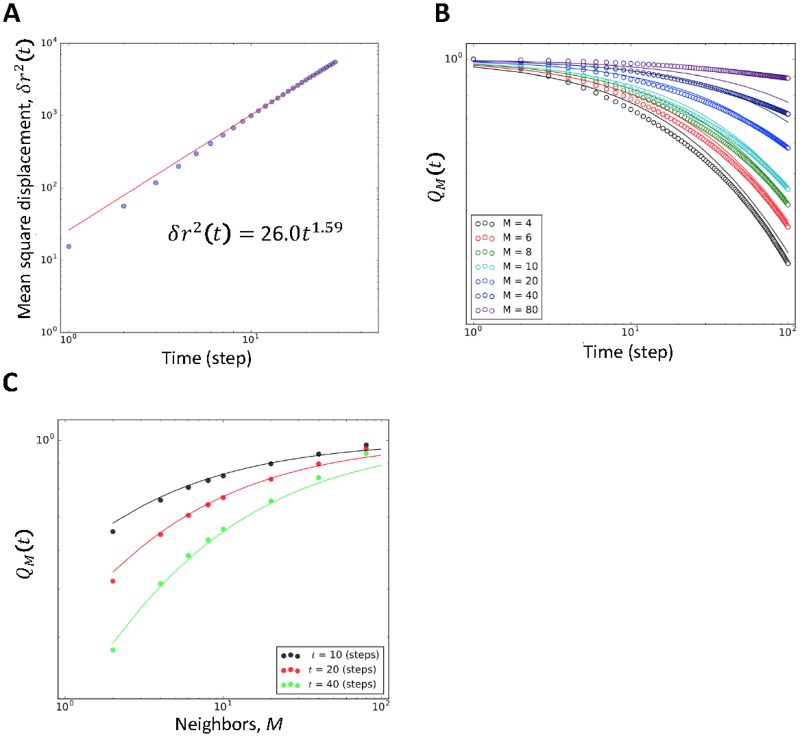
Shuffling behavior inside the flock. (A) Mean square displacement in the center of mass reference frame up to 30 steps. *D* = 26.0 and *α* = 1.59. (B) Neighbors overlap. *Q*_*M*_ against time *t* for groups of 100 individuals. Full lines represent Eq (6) for 0.66 (fitted value) with *α*_*m*_ = 1.55 and $\hat{d}=1.67$. (C) Neighbors overlap: *Q*_*M*_ against number of neighbors *M* for 100 individuals. Values are not fitted for high *M* because *M* is too large for the group size (*M* = 80 and *N* = 100).

Our model explains these collective behaviors. Fig 5A shows super-diffusion in a flock, which is described as

$$\delta r^{2}\left(t\right)=\frac{1}{T-t}\frac{1}{N}{\sum_{t_{0}=0}^{T-t-1}\sum_{i=1}^{N}\left[r_{i}\left(t+t_{0}\right)-r_{i}\left(t\right)\right]}^{2},$$ (3)

where ***x***_*i*_(*t*) is the position of agent *i* at time *t*, ***x***_*CM*_(*t*) is the position of the center of the mass of the flock at time *t*, and ***r***_*i*_(*t*) = ***x***_*i*_(*t*) − ***x***_*CM*_(*t*) is the position of agent *i* in the center of the reference frame. We averaged over all *N* and over all time lags of duration *t* in the interval [0, *T*], where *T* is the total time interval (*T* = 2000 steps). The behavior of the mean square displacement *δr*^2^(*t*) is well-described by the following power law

$$\delta r^{2}\left(t\right)=\;Dt^{\alpha },$$ (4)

Where *α* is the diffusion exponent (0–2) and *D* is the diffusion coefficient. The value of *α* is important. If *α* is 1, the process is normal diffusion, and if *α* is >1, the process is super-diffusion (*α* = 2 is purely ballistic diffusion). If the agents undergo super-diffusion collectively, information transfer is much faster than for normal diffusion.

Fig 5A shows that the diffusion exponent of our model is >1 (i.e., super-diffusion). The diffusion exponent, *α*, is 1.55 on average and its value matches the experimental results well (*Plecoglossus altivelis*: 1.34 < *α* <1.73 and starlings: *α =* 1.73; other parameter results for Eq (4) and mutual diffusion are listed in S4 Fig and S1 Table).

This super-diffusive behavior in flocks results in neighbor shuffling. Neighbor shuffling means that each agent’s neighbors change with time. Cavagna and other researchers [17] described this using the time-dependent overlapping neighbors rate *Q*_*M*_

$$Q_{M}\left(t\right)=\frac{1}{N}\sum_{i}\frac{M_{i}\left(t\right)}{M},$$ (5)

where *M*_*i*_(*t*) is the number of neighbors of agent *i* at time *t* + *t*_0_ that share the same neighborhood. members as agent *i*’s member *M* at time *t*_0_. *Q*_*M*_ decreases over time. According to their calculation, the distribution of *Q*_*M*_ along time *t* is well fitted by

$$Q_{M}\left(t\right)={\left(1+c\frac{t^{\alpha_{m}/2}}{M^{1/\hat{d}}}\right)}^{-\hat{d}},$$ (6)

where $\hat{d}$ is an effective dimension, *α*_*m*_ is a mutual diffusion exponent, and *c* is a constant parameter to fit *Q*_*M*_ (the method of obtaining $\hat{d}$ and *α*_*m*_ is shown in S5 and S6 Figs). This function is also satisfied in our results. Agents in our model change position dynamically inside the flock.

### Lévy walk in the flock

The Lévy walk is an optimal searching strategy found widely in nature, from the movement of bacteria to human movements [43–46], especially in foraging strategies. This behavior can be described by

$$P\left(l\right)\;\sim \;l^{-\mu }\;\text{with}\;1<\mu \leq 3,$$ (7)

where *l* is step length and *μ* is a power law exponent. Step length is the cumulative traveling distance between two pausing points. This procedure is based on the intermittent behavior of the Lévy walk. The definition of the pause (i.e., the state of not moving) is the same as the definition used by Murakami and other researchers [19, 43, 44]). Namely, when *dr* > ||***r***_*i*_(*t*) − ***r***_*i*_(*t—*1)||, or when the traveling distance in the center of mass reference frame for one step is smaller than a given threshold, *dr*, agent *i* is regarded as pausing.

**Fig 6:**
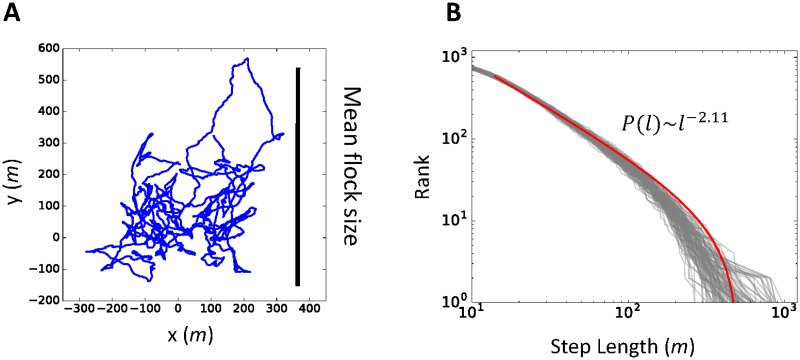
Lévy walk behavior inside flocks. (A) Example of an agent’s trajectory in the center of a mass reference frame for 4000 steps. The vertical bar is the mean flock size during the simulation. (B) Power law distribution of step lengths. The vertical line is the step length rank for each individual. The gray lines correspond to each individual’s step length for its trajectory. The bold red line is the fitting curve with averaged parameters for the truncated power law function. Details of the statistical tests for the power law are given in S2 and S3 Tables and the Method section.

Fig 6A is an example of one agent’s trajectory in the center of the mass reference frame for a group size of 100. The graph shows that the trajectory has ballistic movements (non- entangled) and entangled lines. The trajectory in Fig 6B resembles the trajectories of *Plecoglossus altivelis* in Ref. [19]. This observation is also confirmed through our statistical analysis. Fig 6B shows the step length distributions for 100 agents (gray lines), which are similar. The red line in Fig 6B is the fitting curve with the truncated power law (slope: *μ =* 2.11), which is often used to estimate the Lévy walk power law distribution, of averaged data for 100 distributions. S3 Table lists an example of all the statistical tests for 100 agents and averaged results over 20 simulations for 100 agents for other threshold *dr* settings (see detailed discussion in the Method section or Refs. [47, 48]).

Our result of an average value of *μ =* 2.11 (see S2 Table for various parameter settings) matches Murakami’s experimental data well (1.86 < *μ <* 2.33). Furthermore, an exponent value of *μ ≈* 2 is the value for an optimal foraging strategy in a certain environment [20, 21]. The whole trajectory covers the mean flock size in Fig 6A. As Murakami and other researchers suggested [19], the bustling behavior inside the flock is not restricted to a local region, but occurs throughout the group. Thus, the agents in our model can communicate with each other in the most efficient way.

### Local equilibrium of the flock

We have shown that our model can produce the noisy behavior of individuals. However, the highly ordered macroscale behavior must be connected to the noisy microscale behavior. To bridge this gap, we use Mora and other researcher’s findings [42]. According to their study, parallel processing of different time scale events for micro- and macroscopic behavior explains the apparent paradox of an ordered state being based on a disordered state. They defined two time scales: the alignment time scale, which corresponds to an individual’s behavior over short time scales; and the neighbor exchange time scale, which corresponds to the network (connected topological neighborhood] rearrangement time as a whole flock. We use the name neighbor exchange time instead of using network rearrangement time, following [42].

The alignment time is the relaxation time that eliminates the effect of divergent modes because of the Goldstone theorem (see Ref. [42] for a detailed discussion). The alignment time is approximately *τ*_relax_ = (*Jn*_*c*_)^−1^, where *J* is the overall interaction strength and *n*_*c*_ is the number of nearest neighbors of each agent. This formula is based on an approximation assuming that the group is in a state of high polarity, *O*_*p*_ *≈* 1. Therefore, this method must be applied carefully to our model. We discuss the validity of using this method in the Method section.

The neighbor exchange time is defined as the characteristic decay time of the autocorrelation function *C*_network_(*t*) = ∑_*ij*_*n*_*ij*_(*t*_0_)*n*_*ij*_(*t* + *t*_0_), where $n_{ij}(t)\;=\;e^{{-k}_{ij\;}(t)/n_{c}}$. Here *k*_*ij*_(*t*) is the topological distance at *t*, given as the time-dependent rank of agent *j* among agent *i*’s neighbors ranked by distance. This function decays exponentially, *C*_network_(*t*) ≈ *C*_0_ exp(−t/*τ*_network_) (see S7 Fig).

The relationship between *τ*_relax_ and *τ*_network_ is crucial. When *τ*_relax_ ≈ *τ*_network_, the flock shows only highly polarized behaviors without any rapid shuffling of neighbors in the flock. When *τ*_relax_ ≤ *τ*_network_, the flock shows swarming because the network arrangement is faster than the local alignment speed. The most interesting case is when *τ*_relax_ ≪ *τ*_network_, which is an adiabatic system or local equilibrium. The alignment speed is much faster than the network arrangement speed. This gap between the two time scales enables the flock to have consistent bridging between highly ordered macroscopic behavior and disordered microscopic behavior.

**Fig 7:**
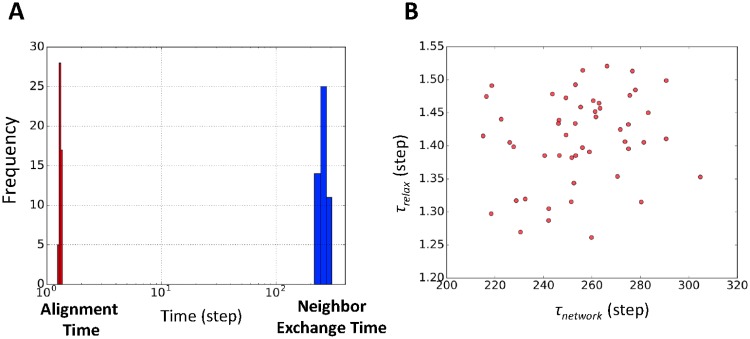
Local equilibrium in flocks. (A) Histogram of alignment time *τ*_relax_ (red bars) and neighbor exchange time *τ*_network_ (blue bars). The vertical line is the frequency for 50 simulations. Alignment time 1/*Jn*_*c*_ is almost equal to 1 step in our simulation where *n*_*c*_ = 5. (B) The scatter plot of *τ*_relax_ versus *τ*_network_ shows no correlation (Pearson correlation coefficient: 0.02).

Fig 7A shows our results. About 200 ordered differences of the two time scales (average of *τ*_relax_ ≈ 180*τ*_network_) are observed in our model. The size of this gap in our model is similar to Mora’s experimental results (*τ*_relax_ ≈ 100*τ*_network_). In addition, we found that there is no correlation between *τ*_relax_ and *τ*_network_, as Mora and other researchers found (Fig 7B; Pearson correlation coefficient: 0.02).

## Discussion

Our model is based on reconsidering attraction and alignment, which most researchers have used for flocking models. We re-interpreted attraction and alignment so that they differ in their time scales, rather than in their fundamental nature. Each agent uses several different time scales through short-term and long-term prediction. The time scale for each agent is heterogeneous in our model because each agent uses different time scale predictions (*τ* and *T*).

The circle *C*, which reflects both short-term and long-term prediction, plays an important role in our model. The interval *I* on circle *C* never gives only one direction, but provides a bundle of directions (movement possibilities) for each agent. As we can see in Fig 4, the ratio between the maximum velocity *V* and the repulsion radius *R* plays a definitive role for various collective behaviors rather than the noise strength. Therefore, making the interval on *C* is the main driving force producing the various properties that we have seen in this study. Note this is not the same as stochastic model with certain probabilistic distributions [49, 50], observed in stochastic differential equation, because our model never gives any probabilistic distributions in advance.

The dots on the circle in our model reflect various time scale events. Having various possible movements means that the agent’s movement is different from computing one specific direction by applying multiple forces (e.g., attraction and alignment) because the covering interval, Cov(*I*) only gives each agent a set of choices. Neighbors’ directions of movement can only restrict an agent’s movement to a certain region no matter how refined their space division ability *p* is. This restriction for direction selection is weaker than in previous flocking models, and it allows our model to exhibit super-diffusion and the Lévy walk.

This type of interaction resembles Gunji and other researcher’s mutual anticipation model [51–53]. The agents in their model also have many possible transition states, although their anticipation method is unique. Gunji and other researchers suggest that having many potential vectors (possible movements) produces a group with high cohesion despite the high noise intensity. The rule that the agent has many possible movements may be key for future models attempting to produce collective ordering while shuffling individual’s positions.

The coexistence of different time scales leads to local equilibrium in our model. Local equilibrium (an adiabatic system) resembles Harken’s synergetics theory [54, 55], known as the enslaving principle, which says that a fast system is directly affected by a slow system. In our flocking model, the alignment is the relatively fast system and the neighbor exchange (or network rearrangement) is the relatively slow system (*τ*_relax_ ≪ *τ*_network_). The theory suggests that network dynamics in the flock directly affects local alignment dynamics, and a time scale gap is necessary to achieve self-organization in the flock. This theory also supports our intuitive understanding of the behavior, as a collective having one mind [4].

Finally, we discuss possible future developments for our model.

*Extension of the model to three dimensions*: Our model extends easily to three dimensions. Some studies have reproduced the coexistence of schooling and milling; however, as we have discussed before, the parameter region for coexistence is very small. The main problem of these studies, we think, is the difficulty of extending to higher dimensions because they use the relative orientation heading for their model and analysis [25, 28]. The relative orientation heading is the angle between two heading directions. The problem emerges when considering the intersection of a bundle of heading directions. This is only valid in two-dimensional space because three-dimensional space has torsions. However, in our model, we simply replace the interaction circle with an interaction sphere and create a spherical cap-shaped covering function for three dimensions.

*Find other prediction methods and other neighborhood shapes*: We introduced the alignment (I) and anticipation (II) prediction methods. Alignment prediction produces Boid-like and SPP-like behavior and anticipation prediction matches many empirical results well. There are more possible prediction methods, which would change the behavior in the model. In addition, different neighborhood shapes may also change the behavior [27, 30]. For example, Sonoda and other researchers [30] found that changing the interaction range from a circle to an ellipse created a long-lasting torus (milling) structure. In our model, although agents formed a torus structure, it lasted for a shorter time than schooling and swarming. Using a different neighborhood shape may make the flock torus structure last longer.

## Methods

### Unit length

We define the unit length as the norm of a unit velocity vector and its indication as *m*.

### Parameter setting

The parameters are as in Table 1 unless indicated otherwise. The ratio between the repulsion radius and the maximum velocity is the most definitive factor for our model because, as we have suggested in the manuscript, collisions (or repulsion) in flock largely depend on an agent’s velocity. In our setting, the ratio between them is fixed at 7/4. Considering the repulsion radius as an agent’s body size, the speed would not be very high compared with its body size. In the Supporting Information, we discuss three ratio values do not affect our result and suggest the possibility that a low ratio would not be good for our analysis.

**Table 1:** Parameters for Figs 4 to 7.

Parameter	Values	Symbol	Unit
Number of agents	100	*N*	None
Maximum velocity	7	*V*	*m* per step
Repulsion radius	4	*R*	*m* per step
Noise strength	0.2	*ζ*	Degrees (rad)
Constant parameter	10	*c*	*m*
Time	1	*t*	Steps

### Threshold value *dr* for Lévy walk

We set the threshold parameter *dr* for each trial to obtain enough step length data: *dr* = 0.8*D*, where *D* is $\frac{1}{NT}\sum_{i\;=\;1}^{N}\sum_{t\;=\;0}^{T-1}\left(\left(r_{i}(t\;+\;1)-\;r_{i}(t)\right)\right)$ and *T* = 9000. The parameter *dr* determines the flock’s average travelling distance during the whole process in the center of mass of the reference frame. We examined three cases (*dr* = 0.9*D*, 0.8*D* and 0.7*D*) to demonstrate the threshold parameter-independence of our results.

### Validity of the approximation for *τ*_relax_ ≈ 1/*Jn*_*c*_

Mora’s approximation for the relaxation time can apply at high polarities, that is, *O*_*p*_ *≈* 1. To apply this method to our flocking model, the method requires modifications because the noisy behaviors in our model prevent stable high-polarity behaviors from being obtained. To confirm the validity of the method for our results, we used two treatments: (i) creating a highly ordered group with *O*_*p*_ > 0.9, and (ii) omitting the out values that come from highly noisy behavior. These are discussed in more detail below. Compared with *τ*_relax_, obtaining *τ*_network_ does not need such careful treatment. All the graph curves apparently satisfy exponential approximations (S7 Fig).

1. *Setting high-velocity agents* (*V* = 20 *and R* = 2): Under this condition, the average *O*_*p*_ is 0.93. The approximation of the overall interaction strength, *J*, is valid under this condition. This condition rarely happens in real flocks, especially in two dimensions, because the agent’s speed (*V*) is 10 times faster than its body length (*R*). This condition is only used to examine the validity of the approximation.
2. *Out values*: The out value is far from the average of a series of trials (2000 steps). In S8 Fig and S4 Table, the average *J* values with no out values are around 0.1. However, out values with very low (<<0.1) or even negative *J* values occur because the average *O*_*p*_ is far from 1.0 (e.g. *O*_*p*_ *≈* 0.5, when *V* = *R* = 4). Fortunately, these values are rare in our model, occurring at most about once in 2000 steps (*V* = 4 and *R* = 4). Furthermore, we have no out values at all when *V* = 20 and *R* = 2. This proves that Mora’s approximation works when *V* = 20 and *R* = 2. Therefore, we expect that the approximation is valid for most cases.

## Supporting information

S1 Fig Frequency distribution of *s* in the anticipation method. The value of *s* indicates how long an agent refers to its neighbor’s past movement in terms of a number of steps. Each bar corresponds to one parameter set: *V*/*R* = 4/4 (blue), 7/4 (green), 10/4 (red). The graph shows that in our setting *s*-values concentrate at the minimum, that is, most agents refer back one step before movement.
(PDF)

S2 FigTwo examples of the covering function when *p* = 32 and 8.The light blue band is any interval *I* on the edge of the neighborhood. Light red bands are $\text{Cov}\left(I\right)=\cup_{a\in \left[I\right]}{\tilde{c}}_{a}$ for each *p*. The covered intervals tend to be larger when *p* is low.(PDF)

S3 Fig Maximum and minimum values for O_*p*_ and O_*T*_. (A) (B) Average maximum values for *O*_*p*_ and *O*_*T*_. The horizontal axis is maximum velocity *V* with repulsion radius *R* = 2 and number of individuals *N* = 100. The value of *O*_*T*_ tends to be high in the low velocity regions. (C) (D) Average minimum values for *O*_*p*_ and *O*_*T*_. The horizontal axis is maximum velocity *V* with repulsion radius *R* = 2 and number of individual *N* = 100. The value of *O*_*P*_ is less than 0.3 in the high velocity regions, although the average *O*_*p*_ values are more than 0.9 in Fig 2B.
(PDF)

S4 FigNormal diffusion and mutual diffusion for each parameter set.(A) Graphs of the super-diffusion for our results on other parameters (*V* = 4 and 10 with *R* = 4). (B) Graphs of mutual diffusion $\delta r_{m}^{2}$ for our results with other parameters (*V* = 4 and 10 with *R* = 4), where $\delta r_{m}^{2}\;=\;\frac{1}{T-t}\frac{1}{N}\sum_{t_{0}\;=\;0}^{T-t-1}\sum_{i\;=\;1}^{N}{\left[||s_{ij}(t+t_{0})||-||s_{ij}(t_{0})||\right]}^{2}$ and ***s***_*ij*_(*t*) = ***r***_*i*_^*t*^–***r***_*j*_^*t*^ (agent *j* is the nearest neighbor of *i* at time *t*_0_). Mean displacement $\delta r_{m}^{2}$ approximately fits power law function $\delta r_{m}^{2}\;=\;D_{m}t^{\alpha_{m}}$.(PDF)

S5 FigNeighbor overlap for *M* and *t*.(A) (B) Graphs of the neighbor overlap value *Q*_*M*_(*t*) along the number of neighbors *M* for our results with other parameters (*V* = 4 and 10 with *R* = 4). (C) (D) Graphs of the neighbor shuffling along time *t* for our results with other parameters (*V* = 4 and 10 with *R* = 4). Both full lines represent Eq (6) in the main manuscript with 0.073 (fitted value), where *α*_*m*_ = 1.49 and $\hat{d}\;=1.71$ for *V* = 4 and with 0.066 (fitted value), where *α*_*m*_ = 1.57 and $\hat{d}\;=1.65$ for *V* = 10.(PDF)

S6 FigNumber of agent’s neighbors *M* as a function of radius *R* of the sphere containing them.The scatter is fitted by $M\;=\;aR^{\hat{d}}$ up to *M* = 30 when *N* = 50, *V* = 7, and *R* = 4. In this figure, *a* = 0.02, $\hat{d}\;=1.67$, and *R*^2^ = 0.998. $\hat{d}\;$ gives effective dimensions for fitting Eq 6.(PDF)

S7 Fig Example of an exponential function *C*_network_(*t*) ≈ *C*_0_exp(−*t*/*τ*_network_) for 50 samples with *V* = 7 and *R* = 4. All the curves decay exponentially.
(PDF)

S8 Fig Histogram of interaction strength *J* for each parameter set for 50 samples. The value of *J* is low for low velocities because of the collision effects. The lowest *J* value is around 0.11. Considering *n*_*c*_ = 5 (median of the number of neighbors), *τ*_relax_ is less than 2 (steps) at most. In particular, for *V* = 7 and *R* = 4, *τ*_relax_ ranges from 1.33 (steps) to 1.53 (steps).
(PDF)

S1 Table Averaged results for running the simulation 20 times for *N* = 100. (PDF)

S2 TableAveraged results for running the simulation 20 times for different parameter sets and different threshold parameters.The exponentials of power law *μ* are around 2. The pass rate is the average pass rate for the Kolmogorov–Smirnov (KS) test [36–40]. We use the truncated power law function to fit our flocking data [37, 38]. The function of the truncated power law function is $f(x)\;=\;(\mu -1)/(x_{min}^{1-\mu }-x_{max}^{1-\mu })x^{-\mu }$, where *μ* is the power law exponent, *x*_*min*_ is the start of the tail of a series of the trial, and *x*_*max*_ is the maximum value of a series of the trial. A pass rate of 1 means that the agent’s step length distribution in a flock can be fitted by the truncated power law distributions. For *V* = 4, the step length distributions tend to fail the KS test because of the collision effect among agents in the flock. The collision effect means that the agents at low velocities tend to have high collision probabilities. Because the repulsion force caused by collision makes an agent turn away from its neighbors, the ballistic movements of agents in the flock disappear.(PDF)

S3 Table Example of the power law test for all the flock members (all the distributions in Fig 6B and threshold *dr* = 0.8*D*). The test followed Clauset’s methods [39]. If *p* > 0.1, then the power law assumption cannot be rejected. All the agents pass the KS test. In addition to the KS test, we also used the Akaike Information Criterion (AIC) to examine whether the truncated power law graph can be distinguished from the exponential function *f*(*x*) = *λ*exp(−*λ*(*x* − *x*_*min*_)), where *λ* is the exponential parameter [36]. If the AIC value is close to 1, the obtained graphs are more likely to follow the truncated power law rather than the exponential power law.
(PDF)

S4 Table Averaged results for running the simulations 50 times for *N* = 100. From the left, average gap between *τ*_network_ and *τ*_relax_, Pearson correlation coefficient (PCC) and its *p* value, which show no correlation for all parameter sets, and average out value through 2000 steps in a simulation series.
(PDF)

S1 Text Algorithm description and the definition of symbols and functions. (PDF)

S1 Movie Example of the simulation of our model using alignment prediction methods for *N* = 50. (MP4)

S2 Movie Example of the simulation of our model using the anticipation methods for *N* = 50. (MP4)
