# Supplementary figures and images for: Entangled time in flocking: Multi-time-scale interaction reveals emergence of inherent noise
Source: PLoS One. 2018 Apr 24;13(4):e0195988. doi: 10.1371/journal.pone.0195988 (PMC5915279; doi:10.1371/journal.pone.0195988)

**S1 Fig**

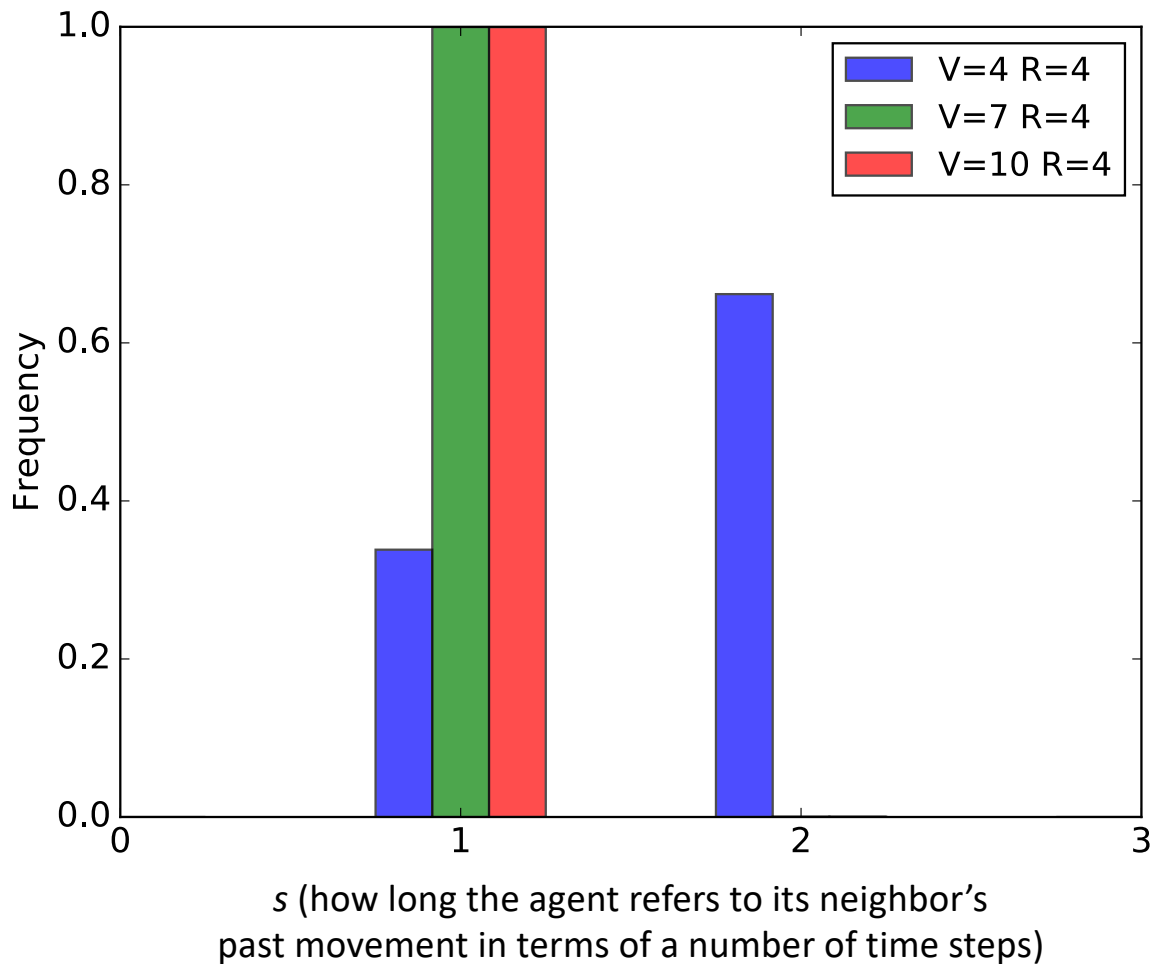

Supplement: S1 Fig — The value of s indicates how long an agent refers to its neighbor’s past movement in terms of a number of steps. Each bar corresponds to one parameter set: V/R = 4/4 (blue), 7/4 (green), 10/4 (red). The graph shows that in our setting s-values concentrate at the minimum, that is, most agents refer back one step before movement. (PDF) [file pone.0195988.s001.pdf]

S2 Fig

32-division

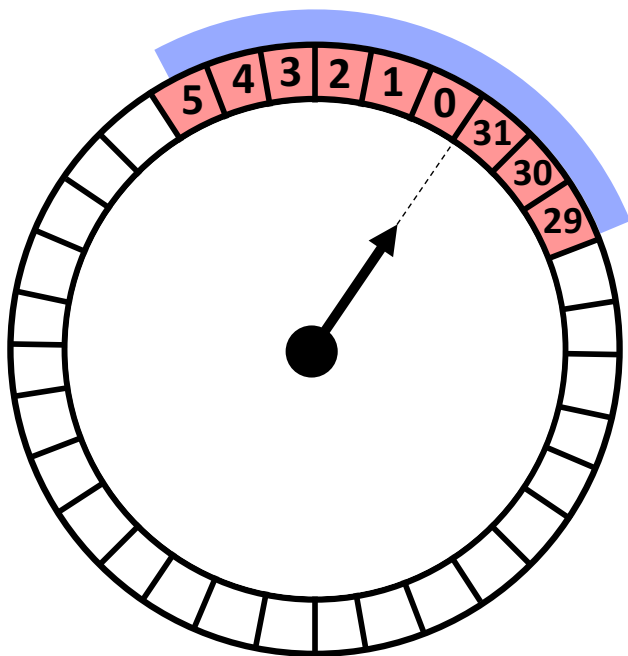

8-division

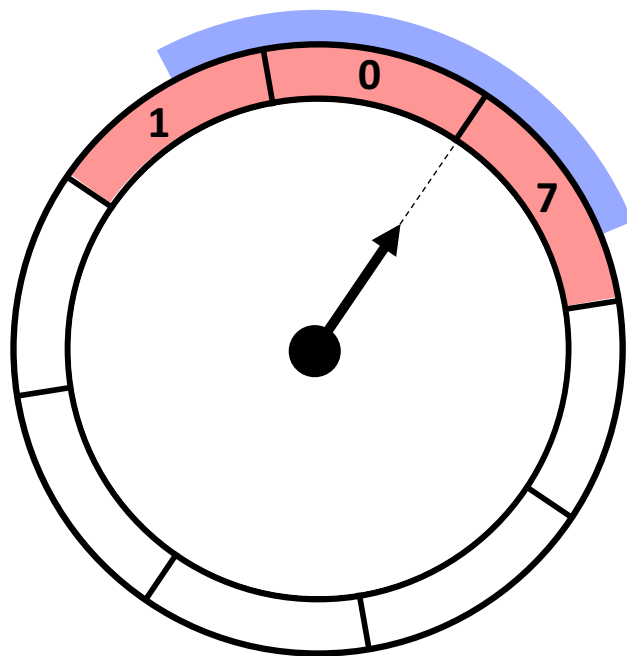

$$[I] = \{0, 1, 2, 3, 4, 5, 29, 30, 31, 32\}$$

$$[I] = \{0, 1, 7\}$$

$$\text{Cov}(I) = \cup_{a \in [I]} \tilde{\mathbf{c}}_a:$$

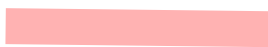

$I :$

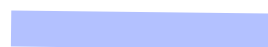

Supplement: S2 Fig — The light blue band is any interval I on the edge of the neighborhood. Light red bands are Cov(I)=∪a∈[I]c˜a for each p. The covered intervals tend to be larger when p is low. (PDF) [file pone.0195988.s002.pdf]

S3 Fig

A

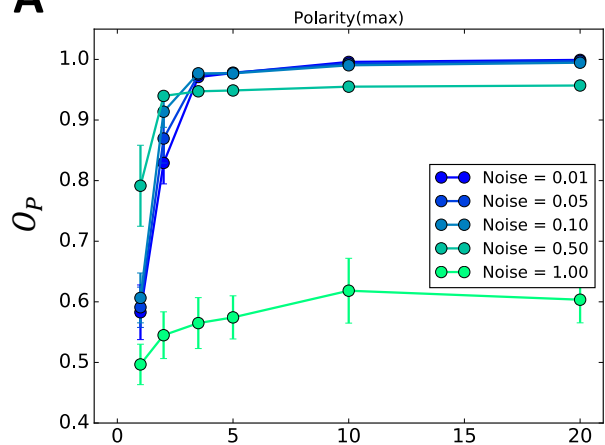

B

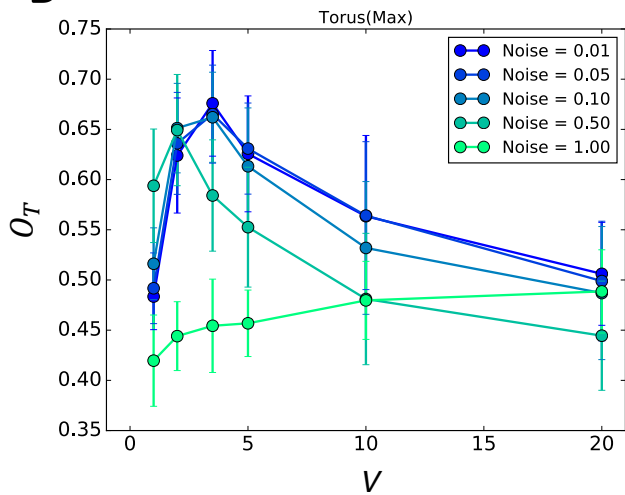

C

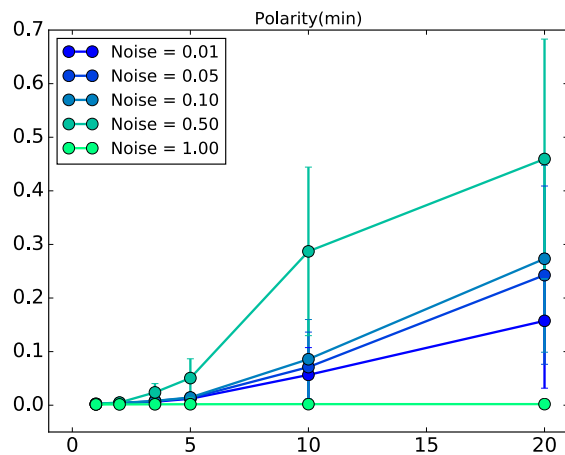

D

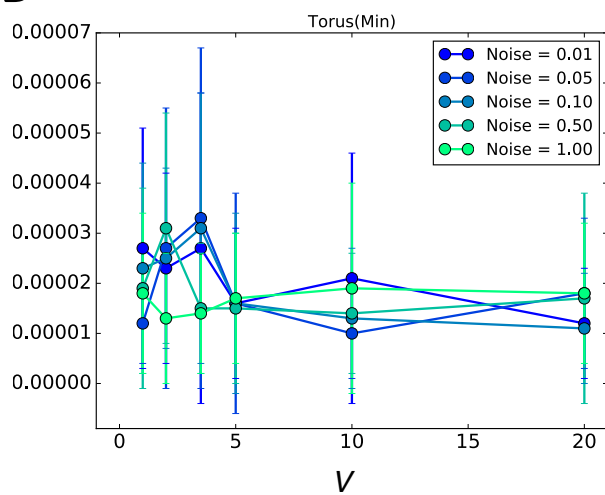

Supplement: S3 Fig — (A) (B) Average maximum values for Op and OT. The horizontal axis is maximum velocity V with repulsion radius R = 2 and number of individuals N = 100. The value of OT tends to be high in the low velocity regions. (C) (D) Average minimum values for Op and OT. The horizontal axis is maximum velocity V with repulsion radius R = 2 and number of individual N = 100. The value of OP is less than 0.3 in the high velocity regions, although the average Op values are more than 0.9 in Fig 2B. (PDF) [file pone.0195988.s003.pdf]

# S4 Fig

**A**

$V = 4$  and  $R = 4$

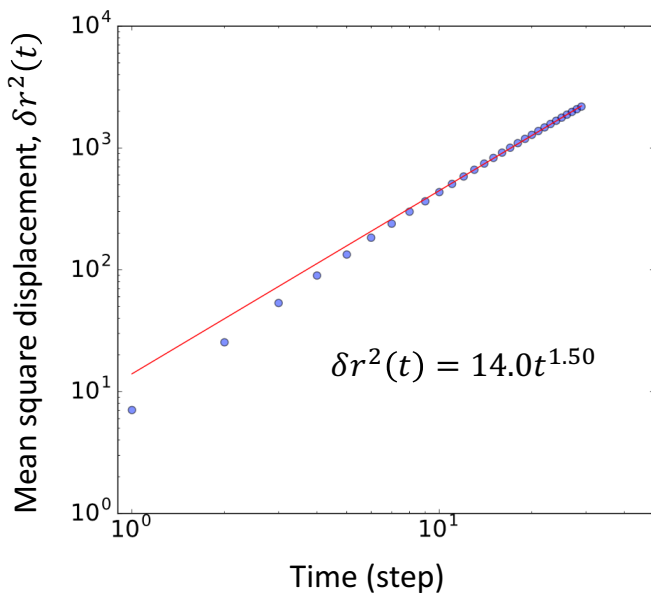

$V = 10$  and  $R = 4$

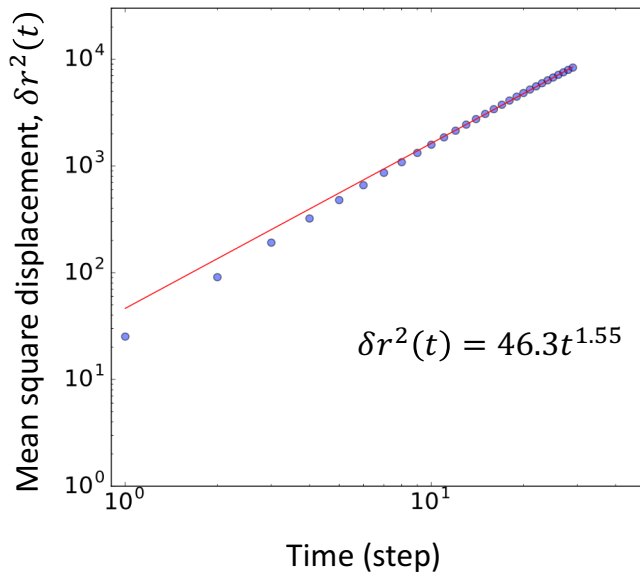

**B**

$V = 4$  and  $R = 4$

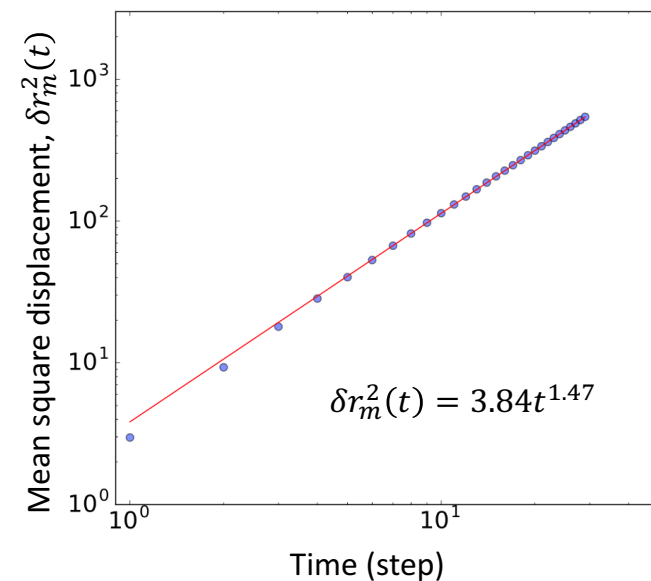

$V = 10$  and  $R = 4$

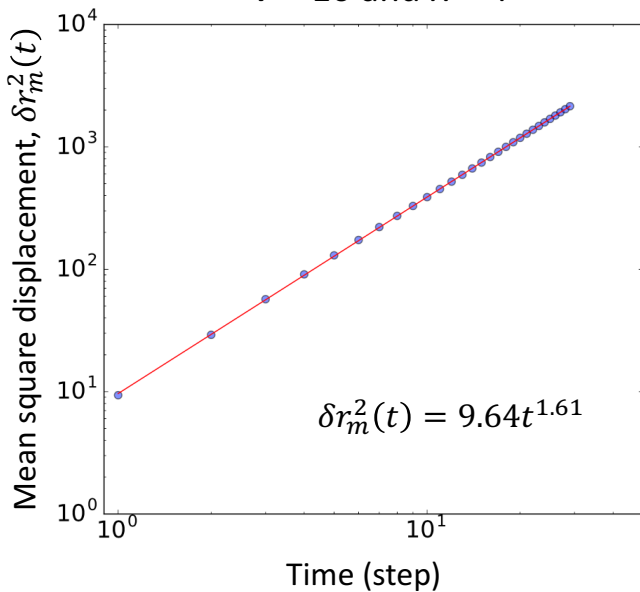

Supplement: S4 Fig — (A) Graphs of the super-diffusion for our results on other parameters (V = 4 and 10 with R = 4). (B) Graphs of mutual diffusion δrm2 for our results with other parameters (V = 4 and 10 with R = 4), where δrm2=1T-t1N∑t0=0T-t-1∑i=1N[||sij(t+t0)||-||sij(t0)||]2 and sij(t) = rit–rjt (agent j is the nearest neighbor of i at time t0). Mean displacement δrm2 approximately fits power law function δrm2=Dmtαm. (PDF) [file pone.0195988.s004.pdf]

**S5 Fig**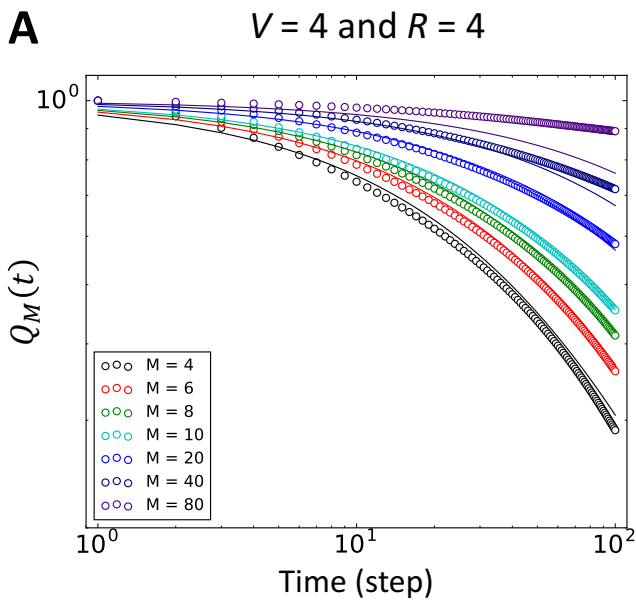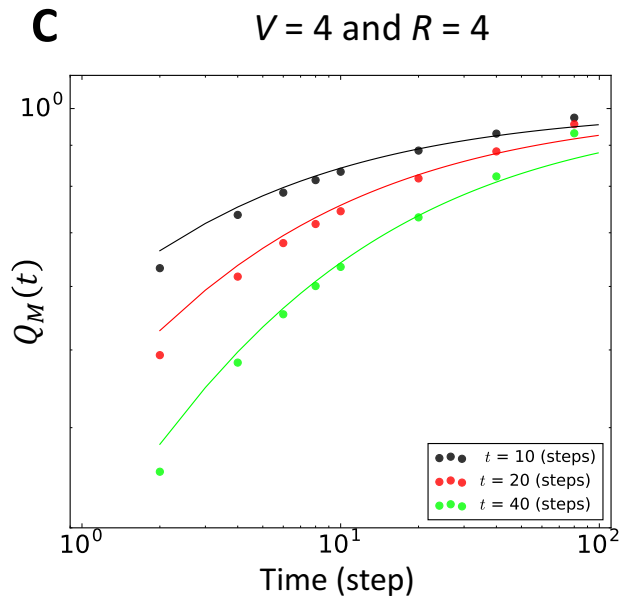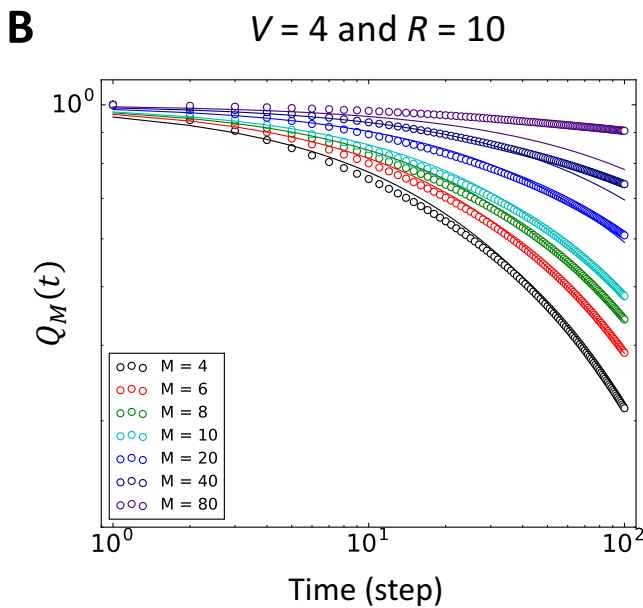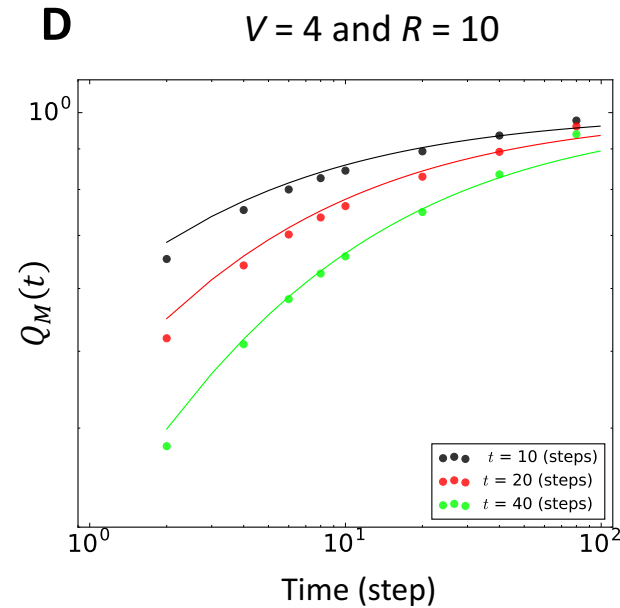

Supplement: S5 Fig — (A) (B) Graphs of the neighbor overlap value QM(t) along the number of neighbors M for our results with other parameters (V = 4 and 10 with R = 4). (C) (D) Graphs of the neighbor shuffling along time t for our results with other parameters (V = 4 and 10 with R = 4). Both full lines represent Eq (6) in the main manuscript with 0.073 (fitted value), where αm = 1.49 and d^=1.71 for V = 4 and with 0.066 (fitted value), where αm = 1.57 and d^=1.65 for V = 10. (PDF) [file pone.0195988.s005.pdf]

**S6 Fig**

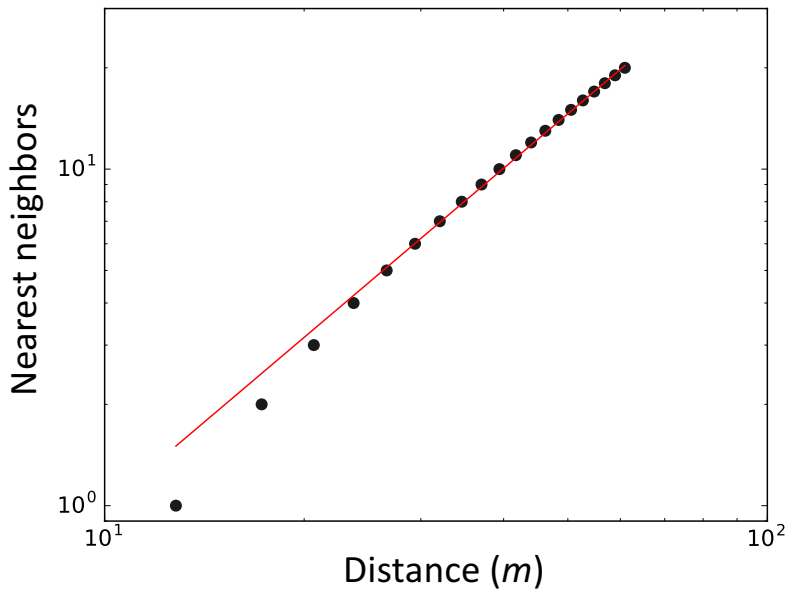

Supplement: S6 Fig — The scatter is fitted by M=aRd^ up to M = 30 when N = 50, V = 7, and R = 4. In this figure, a = 0.02, d^=1.67, and R2 = 0.998. d^ gives effective dimensions for fitting Eq 6. (PDF) [file pone.0195988.s006.pdf]

**S7 Fig**

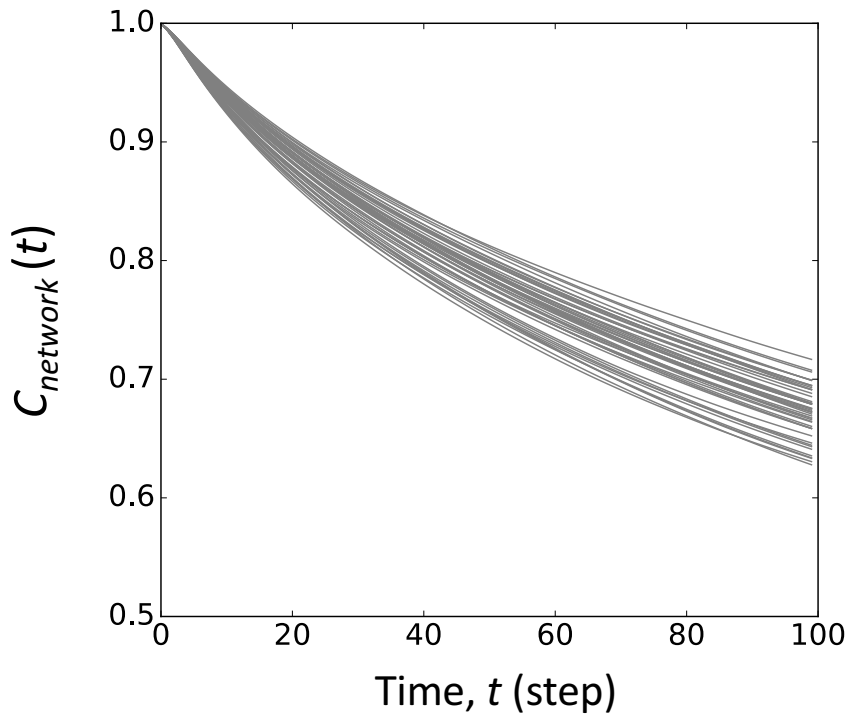

Supplement: S7 Fig — All the curves decay exponentially. (PDF) [file pone.0195988.s007.pdf]

**S8 Fig**

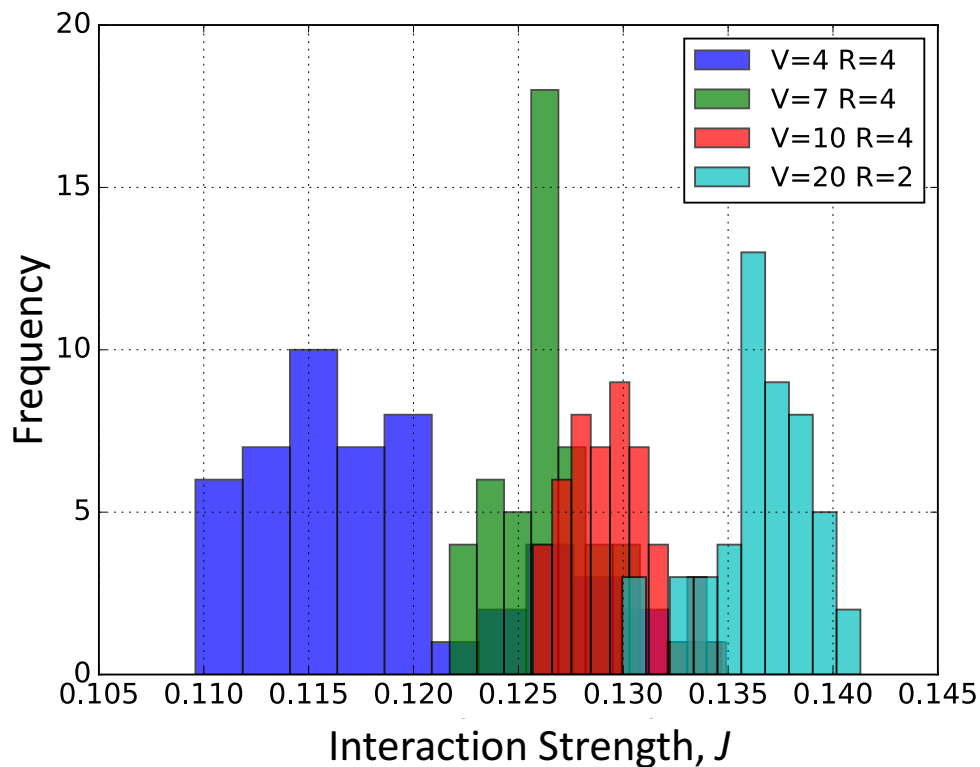

Supplement: S8 Fig — The value of J is low for low velocities because of the collision effects. The lowest J value is around 0.11. Considering nc = 5 (median of the number of neighbors), τrelax is less than 2 (steps) at most. In particular, for V = 7 and R = 4, τrelax ranges from 1.33 (steps) to 1.53 (steps). (PDF) [file pone.0195988.s008.pdf]
