# Supplementary material for: Entangled time in flocking: Multi-time-scale interaction reveals emergence of inherent noise
Source: PLoS One. 2018 Apr 24;13(4):e0195988. doi: 10.1371/journal.pone.0195988 (PMC5915279; doi:10.1371/journal.pone.0195988)
Supplement: S1 Table — (PDF) [file pone.0195988.s009.pdf]

S1 Table.

	Normal diffusion		Mutual diffusion	
	D	α	D_m	α_m
V = 4 and R = 4	15.0 ± 0.97	1.49 ± 0.03	4.20 ± 0.30	1.49 ± 0.02
V = 7 and R = 4	26.3 ± 1.60	1.55 ± 0.04	6.01 ± 0.52	1.56 ± 0.05
V = 10 and R = 4	42.7 ± 2.99	1.54 ± 0.05	9.36 ± 0.95	1.57 ± 0.05
