# Supplementary material for: Entangled time in flocking: Multi-time-scale interaction reveals emergence of inherent noise
Source: PLoS One. 2018 Apr 24;13(4):e0195988. doi: 10.1371/journal.pone.0195988 (PMC5915279; doi:10.1371/journal.pone.0195988)
Supplement: S2 Table — The exponentials of power law μ are around 2. The pass rate is the average pass rate for the Kolmogorov–Smirnov (KS) test [36–40]. We use the truncated power law function to fit our flocking data [37, 38]. The function of the truncated power law function is f(x)=(μ-1)/(xmin1-μ-xmax1-μ)x-μ, where μ is the power law exponent, xmin is the start of the tail of a series of the trial, and xmax is the maximum value of a series of the trial. A pass rate of 1 means that the agent’s step length distribution in a flock can be fitted by the truncated power law distributions. For V = 4, the step length distributions tend to fail the KS test because of the collision effect among agents in the flock. The collision effect means that the agents at low velocities tend to have high collision probabilities. Because the repulsion force caused by collision makes an agent turn away from its neighbors, the ballistic movements of agents in the flock disappear. (PDF) [file pone.0195988.s010.pdf]

S2 Table.

	$dr = 0.9D$			
	x_{min} (m)	x_{max} (m)	μ	Pass rate (%)
V = 4 and *R* = 4	18.2 ± 0.70	215 ± 14.8	2.44 ± 0.04	0.80 ± 0.07
V = 7 and *R* = 4	18.6 ± 0.77	498 ± 59.5	2.27 ± 0.03	1.00 ± 0.00
V = 10 and *R* = 4	21.3 ± 0.89	669 ± 151	2.29 ± 0.03	1.00 ± 0.00
	$dr = 0.8D$			
	x_{min} (m)	x_{max} (m)	μ	Pass rate (%)
V = 4 and *R* = 4	16.2 ± 0.74	240 ± 17.3	2.26 ± 0.03	0.91 ± 0.04
V = 7 and *R* = 4	14.2 ± 0.40	531 ± 70.0	2.11 ± 0.03	1.00±0.00
V = 10 and *R* = 4	16.7 ± 0.71	703 ± 160	2.15 ± 0.03	1.00±0.00
	$dr = 0.7D$			
	x_{min} (m)	x_{max} (m)	μ	Pass rate (%)
V = 4 and *R* = 4	14.3 ± 0.96	269 ± 19.3	2.05 ± 0.03	0.91 ± 0.03
V = 7 and *R* = 4	10.6 ± 0.49	567 ± 71.7	1.96 ± 0.03	1.00±0.00
V = 10 and *R* = 4	12.9 ± 0.65	744 ± 167	2.01 ± 0.03	1.00±0.00
