# Supplementary material for: Entangled time in flocking: Multi-time-scale interaction reveals emergence of inherent noise
Source: PLoS One. 2018 Apr 24;13(4):e0195988. doi: 10.1371/journal.pone.0195988 (PMC5915279; doi:10.1371/journal.pone.0195988)
Supplement: S3 Table — The test followed Clauset’s methods [39]. If p > 0.1, then the power law assumption cannot be rejected. All the agents pass the KS test. In addition to the KS test, we also used the Akaike Information Criterion (AIC) to examine whether the truncated power law graph can be distinguished from the exponential function f(x) = λexp(−λ(x − xmin)), where λ is the exponential parameter [36]. If the AIC value is close to 1, the obtained graphs are more likely to follow the truncated power law rather than the exponential power law. (PDF) [file pone.0195988.s011.pdf]

Number of data	X_{min}	X_{max}	Scaling parameter μ	p value	AIC value
1092	9.4	364.1	1.92	0.107	1
1122	10.3	429.7	2.01	0.125	1
1128	17.9	389.3	2.23	0.113	1
1113	12.7	617.7	2.06	0.120	1
1162	12.0	320.2	2.08	0.108	1
1068	11.6	457.1	2.07	0.140	1
1074	12.9	550.0	2.09	0.131	1
1123	11.9	396.5	2.07	0.115	1
1094	17.3	857.4	2.31	0.114	1
1043	10.7	530.0	1.94	0.112	1
1029	12.5	788.1	2.05	0.113	1
1054	16.0	429.5	2.14	0.126	1
1122	13.5	453.7	2.12	0.129	1
1081	13.7	568.9	2.15	0.120	1
1111	13.9	551.4	2.13	0.156	1
1080	19.1	642.9	2.16	0.162	1
1114	13.0	820.1	2.19	0.115	1
1155	13.6	386.6	2.15	0.119	1
1109	13.9	383.2	2.11	0.112	1
1079	13.9	452.5	2.14	0.117	1
1086	13.6	328.7	2.12	0.122	1
1111	13.9	506.1	2.14	0.123	1
1060	13.0	368.4	2.00	0.117	1
1111	16.4	879.8	2.33	0.116	1
1079	14.0	634.8	2.18	0.139	1
1125	12.1	345.7	2.06	0.110	1
1093	14.9	429.1	2.14	0.112	1
1103	13.2	485.4	2.17	0.136	1
1114	14.2	496.8	2.17	0.113	1
1091	20.4	790.5	2.35	0.113	1
1118	12.9	447.0	2.12	0.123	1
1067	15.0	360.4	2.09	0.123	1
1104	13.9	410.7	2.14	0.113	1
1151	14.3	433.5	2.18	0.107	1
1078	14.3	652.0	2.21	0.130	1
1131	15.3	501.2	2.13	0.112	1
1097	15.6	477.3	2.14	0.206	1
1045	11.8	478.9	2.03	0.126	1
1116	13.1	474.8	2.09	0.111	1
1151	12.5	321.5	2.13	0.117	1
1074	19.5	883.7	2.31	0.117	1
1073	19.4	825.4	2.30	0.147	1
1106	11.0	509.2	2.02	0.121	1
1083	11.3	349.0	2.00	0.155	1
1037	15.8	351.0	2.14	0.137	1
1011	12.7	597.7	2.00	0.118	1
1091	13.3	513.7	2.09	0.112	1
998	22.6	700.7	2.27	0.117	1
1145	14.6	357.0	2.19	0.113	1
1055	16.7	493.4	2.12	0.112	1

Number of data	X_{min}	X_{max}	Scaling parameter μ	p value	AIC value
1130	11.3	303.0	2.01	0.122	1
1090	12.4	519.4	2.14	0.108	1
1131	18.8	359.4	2.24	0.118	1
1084	15.0	391.7	2.10	0.107	1
1092	10.8	409.4	1.96	0.126	1
1095	14.3	315.3	2.07	0.126	1
1136	12.1	424.3	2.09	0.109	1
1071	15.0	611.6	2.14	0.148	1
1060	13.2	537.1	2.08	0.130	1
1094	13.5	397.4	2.09	0.131	1
1034	10.7	266.5	1.88	0.118	1
1035	32.0	778.9	2.39	0.155	1
1081	13.4	650.1	2.13	0.124	1
1089	13.7	748.7	2.17	0.110	1
1114	16.1	552.8	2.21	0.114	1
1060	14.9	508.3	2.18	0.173	1
1100	14.4	491.1	2.17	0.117	1
1069	13.6	350.1	2.08	0.123	1
1066	13.5	572.4	2.13	0.120	1
1093	14.9	458.9	2.14	0.110	1
996	17.9	846.1	2.19	0.125	1
1106	11.9	397.6	2.05	0.142	1
1074	10.4	338.8	1.90	0.121	1
1119	13.0	466.3	2.08	0.118	1
1060	13.7	667.7	2.07	0.123	1
1047	12.0	264.2	1.91	0.126	1
1088	11.2	418.0	1.95	0.114	1
1112	18.1	594.1	2.29	0.117	1
1058	15.4	470.2	2.09	0.117	1
1106	14.0	371.0	2.12	0.115	1
1055	16.8	727.4	2.18	0.135	1
1108	14.3	425.4	2.15	0.128	1
1116	10.7	333.9	2.01	0.121	1
1047	12.3	514.3	2.03	0.110	1
1114	14.3	411.6	2.13	0.113	1
1027	11.7	461.0	2.00	0.122	1
1067	14.4	828.5	2.13	0.121	1
1089	13.8	969.6	2.15	0.128	1
1067	14.5	295.7	2.05	0.133	1
1092	11.7	382.2	2.06	0.143	1
1036	14.0	637.6	2.12	0.124	1
1078	14.4	474.0	2.17	0.117	1
1115	18.8	411.3	2.26	0.111	1
1058	15.5	611.0	2.20	0.108	1
1075	13.2	410.3	2.08	0.129	1
1139	14.8	496.4	2.21	0.121	1
1043	19.8	503.3	2.20	0.133	1
1079	14.0	536.3	2.06	0.120	1
1109	16.2	873.0	2.30	0.171	1
1029	12.7	379.1	1.97	0.116	1
