# Supplementary material for: Entangled time in flocking: Multi-time-scale interaction reveals emergence of inherent noise
Source: PLoS One. 2018 Apr 24;13(4):e0195988. doi: 10.1371/journal.pone.0195988 (PMC5915279; doi:10.1371/journal.pone.0195988)
Supplement: S4 Table — From the left, average gap between τnetwork and τrelax, Pearson correlation coefficient (PCC) and its p value, which show no correlation for all parameter sets, and average out value through 2000 steps in a simulation series. (PDF) [file pone.0195988.s012.pdf]

S4 Table.

	$\tau_{\mathrm{network}} = x * \tau_{\mathrm{relax}}$	PCC	*p* value	Out value
V = 4 and *R* = 4	170 ± 14.9	0.16	0.27	1.76 ± 1.44
V = 7 and *R* = 4	193 ± 16.1	0.02	0.88	0.18 ± 0.44
V = 10 and *R* = 4	201 ± 16.3	0.17	0.28	0.08 ± 0.34
V = 20 and *R* = 2	212 ± 17.6	0.06	0.68	0.00 ± 0.00
