# Supplementary material for: Entangled time in flocking: Multi-time-scale interaction reveals emergence of inherent noise
Source: PLoS One. 2018 Apr 24;13(4):e0195988. doi: 10.1371/journal.pone.0195988 (PMC5915279; doi:10.1371/journal.pone.0195988)
Supplement: S1 Text — (PDF) [file pone.0195988.s013.pdf]

Supporting Information

Algorithm Notation

(i) Symbols

Individuals: $\boldsymbol{N} = \{1,\ 2, 3, \ldots, N\}$ and $|\boldsymbol{N}| = N$

Agent's position vector: $\boldsymbol{x}$

Agent's position vector in the center of mass reference frame: $\boldsymbol{r}$

Agent's velocity vector: $\boldsymbol{v}$

Agent's unit velocity vector: $\boldsymbol{u}$

Agent's maximum velocity: V

Agent's direction: φ

Neighbor network: $\boldsymbol{E} = \{(i,j) |\ i, j \in \boldsymbol{N}, i \text{ and } j \text{ are Voronoi neighbors}\}$

Agent i's neighbors: $\boldsymbol{n}_i = \{j |\ j \in \boldsymbol{N}, (i,j) \in \boldsymbol{E} \text{ or } i = j\}$

Repulsion Radius: R

Agent i's neighbors in its repulsion: $\boldsymbol{o}_i = \{j |\ d(\boldsymbol{x}_i, \boldsymbol{x}_j) < R \text{ or } i = j\}$

Unit circle: $C_{\mathbf{u}} = \{\theta | 0 \leq \theta < 2\pi\}$

Noise parameter: ζ

Constant parameter: c

(ii) Function symbols

Let S be a set and X be a set such that $\forall\theta \in X,\ 0 \leq \theta < 2\pi$.

Euclidean Distance function: $d(\boldsymbol{x}, \boldsymbol{y}) = ||\boldsymbol{x} - \boldsymbol{y}||\ (\forall \boldsymbol{x}, \boldsymbol{y} \in \boldsymbol{R}^2)$

Argument of vector $\boldsymbol{v}$: arg($\boldsymbol{v}$)

Mean angle function: $\mathrm{mean}(X) = \arg(\frac{1}{|X|}\sum_{\theta \in X}(\cos(\theta), \sin(\theta)))$

Covering function: $\mathrm{Cov}(X) = \{\theta |\ \min(\{\theta_i - \mathrm{mean}(X) |\ \theta_i \in X\}) \leqq \theta - \mathrm{mean}(X) \leqq \max(\{\theta_i - \mathrm{mean}(X) |\ \theta_i \in X\})\}$

Random function: $Ran(S) = s$ and s is randomly selected from a set S

Quasi-attraction points

Given a set of agent i's neighbors $\boldsymbol{n}_i(t)$ at time t, each quasi-attraction point $P_{\tau_j}(t)(j \in \boldsymbol{n}_i(t))$ is an intersection point where the half line along the direction of neighbor j's crosses agent i's radius $C_i(t) = \boldsymbol{x}_i(t) + \{(r_i(t)\cos(\theta), r_i(t)\sin(\theta)) | 0 \le \theta < 2\pi\}$, where $r_i(t) = \max(\{d(\boldsymbol{x}_i(t), \boldsymbol{x}_j(t)) | j \in \boldsymbol{n}_i(t)\}) + c$. Then, we get $\theta_i(t) = \arg(P_{\tau_j}(t) - \boldsymbol{x}_i(t))$ for each agent's neighbor and its set $\boldsymbol{\theta}_i(t) = \{\theta_1(t), \theta_2(t), \dots, \theta_{|\boldsymbol{n}_i(t)|}(t)\}$.

Quasi-alignment points

1. Alignment prediction

Given a set of agent i's neighbors $\boldsymbol{n}_i(t)$ at time t, each alignment prediction point $Q_{T_j}(t)(j \in \boldsymbol{n}_i(t))$ is $Q_{T_j}(t) = \boldsymbol{x}_j(t) + TV\boldsymbol{u}_j(t)$ (with fixed $T = 300$).

2. Anticipation

Given a set of agent i's neighbors $\boldsymbol{n}_i(t)$, each anticipation point $Q_{T_j}(t)(\forall j \in \boldsymbol{n}_i(t))$ is $Q_{T_j}(t) = \boldsymbol{x}_j(t) + sTV\boldsymbol{u}_j^s(t)$, where $\boldsymbol{u}_j^s(t)$ is a unit velocity vector with an argument of $\varphi_j(t) + d\varphi_j^s(t)$. Natural number s determines how long the agent refers to its past movements, that is, $\boldsymbol{v}_i^s(t) = \boldsymbol{x}_i(t) - \boldsymbol{x}_i(t-s)$ and $\boldsymbol{v}_i^{s-1}(t) = \boldsymbol{x}_i(t-s) - \boldsymbol{x}_i(t-2s)$, then $d\varphi_i^s(t) = \arg(\boldsymbol{v}_i^s(t)) - \arg(\boldsymbol{v}_i^{s-1}(t))$. The prediction point for anticipation is $Q_T = \boldsymbol{x}_i(t) + T \parallel \boldsymbol{v}_i(t) \parallel \boldsymbol{u}_i(t)$, where $\boldsymbol{u}_i(t)$ is a unit vector with argument $\arg(\boldsymbol{v}_i^1(t)) + d\varphi_i(t)$. The symbol ||-|| indicates the vector norm. The value of T is given

by $s*r_1(t)/ \| v_i(t) \|$ where $r_1(t)$ is the radius of the neighborhood at time t. This definition means that enlarging s, that is how long the agent refers to its neighbor's past, enlarges T. The value of s is uniquely determined as the minimum value such that all neighbors' Q_T's lie outside the neighborhood (in Fig S1, we give the graph of the distribution of s, which shows the frequency of s values for our simulations).

Repulsion

Given a set of agent i's neighbors $\boldsymbol{o}_i(t)$ at time t in the repulsion zone, each quasi-attraction point $P_{\tau_j}(t)$ $(\forall j \in \boldsymbol{o}_i(t))$ is an intersection point where the half line along neighbor j's direction crosses agent i's neighborhood $\tilde{C}_i(t) = \boldsymbol{x}_i(t) + \{(R\cos(\theta), R\sin(\theta))\,|\, 0 \le \theta < 2\pi\}$. Then, we get $\theta_i(t) = \arg(P_{\tau_j}(t) - \boldsymbol{x}_i(t))$ for each neighbor and its set $\widetilde{\boldsymbol{\theta}}_i(t) = \{\theta_1(t), \theta_2(t), \ldots, \theta_{|\boldsymbol{o}_i(t)|}(t)\}$.

Outputs for quasi-attraction, quasi-alignment, and repulsion

1. Quasi-attraction and quasi-alignment

Applying the cover functions $\mathrm{Cov}(\boldsymbol{\theta}_i(t))$ and $\mathrm{Cov}(\boldsymbol{\Theta}_i(t))$ for each i at time t, and defining $I_i(t) = \mathrm{Cov}(\boldsymbol{\theta}_i(t)) \cap \mathrm{Cov}(\boldsymbol{\Theta}_i(t))$ and $J_i(t) = \mathrm{Cov}(\boldsymbol{\theta}_i(t)) \cup \mathrm{Cov}(\boldsymbol{\Theta}_i(t))$, if $I_i(t) \neq \emptyset$, then agent i's next direction is $\varphi_i(t+1) = \mathrm{mean}(\varphi_i(t), Ran(I_i(t)))$. If $I_i(t) = \emptyset$, then agent i's next direction is $\varphi_i(t+1) = \mathrm{mean}(\varphi_i(t), Ran(C_{\mathbf{u}} - J_i(t)))$.

2. Repulsion

Applying the cover function $\mathrm{Cov}(\widetilde{\boldsymbol{\theta}}_i(t))$ for agent i at time t, then agent i's next direction is $\varphi_i(t+1) = \mathrm{mean}(\varphi_i(t), Ran(C_{\mathbf{u}} - \mathrm{Cov}(\widetilde{\boldsymbol{\theta}}_i(t))))$.

Noise

The p-division of a unit circle $C_{\mathbf{u}}$, where p is $[2\pi/\zeta]$ and [-] is the floor function, for agent i with direction $\varphi_i(t)$ at time t is $C_{\mathbf{u}} = \bigcup_{a=0}^{p-1} \tilde{\boldsymbol{c}}_a$, where $\tilde{\boldsymbol{c}}_a = \{\theta | \varphi_i(t) + 2\pi a/p \leq \theta < \varphi_i(t) + 2\pi(a+1)/p\}$. Any intervals I in $C_{\mathbf{u}}$ can be partitioned as $\bigcup_{a\in[I]} \tilde{\boldsymbol{c}}_a$, where $[I] = \{a | 0 \leq \forall a < p,\ \tilde{\boldsymbol{c}}_a \cap I \neq \emptyset\}$. Fig S2A shows two images of p-partition along agent i's direction $\varphi_i(t)$.

Algorithm

1. Distribute all agents randomly in a two-dimensional space, which is $100(m) \times 100(m)$ in our model. Each agent also has a random velocity.

2. Each agent checks whether it has its neighbors in its repulsion zone $\tilde{C}_i(t) \neq \emptyset$ or not. If it does, go to 2.1. Otherwise, go to 3.

   2.1. Compute the agent's quasi-attraction points on $\tilde{C}_i(t) = \boldsymbol{x}_i(t) + \{(R\cos(\theta), R\sin(\theta)) | \ 0 \leq \theta < 2\pi\}$ for each agent in $\boldsymbol{o}_i(t) = \{j | d(\boldsymbol{x}_i, \boldsymbol{x}_j) < R\}$ to obtain $\widetilde{\boldsymbol{\theta}}_i(t) = \{\theta_1(t), \theta_2(t), \dots, \theta_{|\boldsymbol{o}_i(t)|}(t)\}$. Make a covering set, $\mathrm{Cov}(\widetilde{\boldsymbol{\theta}}_i(t))$, and determine the agent's next direction, $\varphi_i(t+1) = \mathrm{mean}(\varphi_i(t), Ran(\tilde{c}_{Ran([C_{\mathbf{u}} - \mathrm{Cov}(\widetilde{\boldsymbol{\theta}}_i(t))])}))$.

   2.2. Determine the agent's velocity: $\boldsymbol{v}_i(t+1) = V\cos(\varphi_i(t+1) - \varphi_i(t))$ where V is a maximum velocity.

   2.3. Update the agent's position: $\boldsymbol{x}_i(t+1) = \boldsymbol{x}_i(t) + \boldsymbol{v}_i(t+1)$.

3. Draw Delaunay triangles (i.e., find the Voronoi neighbors) $\boldsymbol{E}_i(t) = \{(i,j) | \ i,j \in \boldsymbol{N}, i \text{ and } j \text{ are Voronoi neighbors}\}$ from agent's distribution to find its directly connected neighbors, $\boldsymbol{n}_i(t) = \{j | (i,j) \in \boldsymbol{E}_i(t)\}$. Obtain the edge of neighborhood $C_i(t) = \boldsymbol{x}_i(t) + \{(r_i(t)\cos(\theta), r_i(t)\sin(\theta)) | \ 0 \leqq \theta < 2\pi\}$ where $r_i(t) = \max(\{d(\boldsymbol{x}_i(t), \boldsymbol{x}_j(t)) | \ j \in \boldsymbol{n}_i(t)\}) + c$ (c is a constant parameter). Compute the agent's quasi-attraction and quasi-alignment points for $\boldsymbol{n}_i(t)$ and get $\boldsymbol{\theta}_i(t)$ and $\boldsymbol{\Theta}_i(t)$ by using the method (I). Make two covering sets ($\mathrm{Cov}(\boldsymbol{\theta}_i(t))$ and $\mathrm{Cov}(\boldsymbol{\Theta}_i(t))$) and take the intersection, $I_i(t) = \mathrm{Cov}(\boldsymbol{\theta}_i(t)) \cap \mathrm{Cov}(\boldsymbol{\Theta}_i(t))$.

   3.1. If $I_i(t) \neq \emptyset$, the agent's next direction is $\varphi_i(t+1) =$

$\mathrm{mean}(\varphi_i(t), Ran(\tilde{\boldsymbol{c}}_{Ran([I_i(t)])}))$.

3.2. If $I_i(t) = \emptyset$, the agent's next direction is $\varphi_i(t+1) = \mathrm{mean}(\varphi_i(t), Ran(\tilde{\boldsymbol{c}}_{Ran([C_{\mathbf{u}} - J_i(t)])}))$.

4. Update the rest of the agent states $\boldsymbol{v}_i(t+1) = V\cos(\varphi_i(t+1) - \varphi_i(t))$ and $\boldsymbol{x}_i(t+1) = \boldsymbol{x}_i(t) + \boldsymbol{v}_i(t+1)$ synchronously. Then update $t \rightarrow t+1$ and return to 2.
